# Head-to-head study of oxelumab and adalimumab in a mouse model of ulcerative colitis based on NOD/Scid IL2Rγ^null^ mice reconstituted with human peripheral blood mononuclear cells

**DOI:** 10.1242/dmm.046995

**Published:** 2021-01-21

**Authors:** Henrika Jodeleit, Paula Winkelmann, Janina Caesar, Sebastian Sterz, Lesca M. Holdt, Florian Beigel, Johannes Stallhofer, Simone Breiteneicher, Eckart Bartnik, Thomas Leeuw, Matthias Siebeck, Roswitha Gropp

**Affiliations:** 1 Department of General, Visceral and Transplantation Surgery, Hospital of the Ludwig-Maximilian-University Munich, Nussbaumstraße 20, 80336 Munich, Germany; 2 Institute of Laboratory Medicine, Hospital of the Ludwig-Maximilian-University Munich, 81377 Munich, Germany; 3 Department of Medicine II, Hospital of the Ludwig-Maximilian-University Munich, Marchioninistraße 15, 81377 Munich, Germany; 4 Immunology and Inflammation Research TA, Sanofi-Aventis Deutschland GmbH, 65926 Frankfurt am Main, Germany

**Keywords:** NOD/Scid IL2Rγ null, NSG mice, Ulcerative colitis, Anti-CD252 antibodies, Inflammatory bowel disease, Oxelumab

## Abstract

This study's aim was to demonstrate that the combination of patient immune profiling and testing in a humanized mouse model of ulcerative colitis (UC) might lead to patient stratification for treatment with oxelumab. First, immunological profiles of UC patients and non-UC donors were analyzed for CD4^+^ T cells expressing OX40 (CD134; also known as TNFRSF4) and CD14^+^ monocytes expressing OX40L (CD252; also known as TNFSF4) by flow cytometric analysis. A significant difference was observed between the groups for CD14^+^ OX40L^+^ (UC: *n*=11, 85.44±21.17, mean±s.d.; non-UC: *n*=5, 30.7±34.92; *P*=0.02), whereas no significant difference was detected for CD4^+^ OX40^+^. CD14^+^ OX40L^+^ monocytes were correlated significantly with T helper 1 and 2 cells. Second, NOD/Scid IL2Rγ null mice were reconstituted with peripheral blood mononuclear cells from UC donors exhibiting elevated levels of OX40L, and the efficacy of oxelumab was compared with that of adalimumab. The clinical, colon and histological scores and the serum concentrations of IL-6, IL-1β and glutamic acid were assessed. Treatment with oxelumab or adalimumab resulted in significantly reduced clinical, colon and histological scores, reduced serum concentrations of IL-6 and reduced frequencies of splenic human effector memory T cells and switched B cells. Comparison of the efficacy of adalimumab and oxelumab by orthogonal partial least squares discrimination analysis revealed that oxelumab was slightly superior to adalimumab; however, elevated serum concentrations of glutamic acid suggested ongoing inflammation. These results suggest that oxelumab addresses the pro-inflammatory arm of inflammation while promoting the remodeling arm and that patients exhibiting elevated levels of OX40L might benefit from treatment with oxelumab.

## Abstract (precis)

**Summary:** The combination of patient profiling and testing in a NOD/Scid IL2Rγ^null^ mouse model of ulcerative colitis (UC) validates oxelumab as a potential therapeutic in UC.

## INTRODUCTION

OX40 (CD134; also known as TNFRSF4) belongs to the family of tumor necrosis factor (TNF) receptors (TNFRs). Expression of OX40 is induced in T cells in response to antigen recognition and other pro-inflammatory factors and is thought to augment antigen-initiated signaling to promote proliferation, differentiation and survival of effector and memory CD4^+^ and CD8^+^ T cells (for review, see Webb et al., 2016). Its cognate ligand OX40L (CD252; also known as TNFSF4) is expressed on antigen-presenting, endothelial and mast cells (Linton et al., 2003; Jenkins et al., 2007; Kashiwakura et al., 2004; Imura et al., 1996). Upon OX40L and OX40 ligation, OX40 oligomerizes and forms signalosomes in membrane lipid microdomains (So et al., 2011). TNF receptor adaptor factors (TRAFs) mediate the interaction with the PI3K/Akt and NFκB pathway, ultimately leading to proliferation, survival and cytokine expression (So and Croft, 2013). OX40-triggered responses control longevity, late proliferation and activation states (Gramaglia et al., 2000; Rogers et al., 2001; Soroosh et al., 2006) and require prior antigen recognition (So et al., 2011). Hence, it has been suggested that close proximity of T cell receptor (TCR)/CD28 and OX40 signalosomes facilitate co-signaling of both pathways. However, the fact that OX40 signaling is independent of TCR co-stimulation by CD28 has led to increasing interest in the capacity of OX40 to break tolerance; indeed, it has been shown that signaling through OX40 breaks peripheral T cell tolerance (Bansal-Pakala et al., 2001). Therefore, agonistic antibodies of OX40 are in development to break tolerance in cancer (Aspeslagh et al., 2016), whereas neutralizing OX40L antibodies (oxelumab) have been developed for treatment of chronic inflammatory diseases, such as asthma. A clinical study has shown reduced serum IgE levels and reduced numbers of airway eosinophils in response to treatment with oxelumab; however, no effect was observed on allergen-induced airway responses (Gauvreau et al., 2014).

Ulcerative colitis (UC) belongs to the chronic inflammatory bowel diseases of unknown etiology. Patients suffer from severe diarrhea, blood loss, abdominal pain and fatigue. The chronic nature of UC leads to a significant and lifelong impact on patients. With the development of monoclonal antibodies, such as infliximab, adalimumab and vedolizumab, treatment of UC has improved greatly; however, the medical need for new approaches remains high. Approximately 40% of all UC patients respond poorly or become refractory to adalimumab or infliximab, requiring alternate treatment regimens. Hence, the medical need to explore other molecular targets is high. Given that OX40 expression has been shown to be increased in lamina propria lymphocytes of inflamed colon and that OX40L expression was increased in endothelium from inflamed colon (Souza et al., 1999), the therapeutic potential of targeting the OX40 pathway was tested in various mouse models of colitis. Either neutralizing antibodies of OX40L or blocking antibodies of OX40 led to amelioration of the disease phenotype (Obermeier et al., 2003; Totsuka et al., 2003; Malmström et al., 2001).

Recently, we have established immune profiles of UC and non-UC patients to obtain a better understanding of the dynamics of the inflammatory processes, to define the subtypes of UC and to attempt to stratify patients for certain therapeutics (Föhlinger et al., 2016; Jodeleit et al., 2017, 2020a). These studies suggest at least two inflammatory conditions: a pro-inflammatory condition driven by T helper (TH)1 and TH2 cells, and a remodeling condition signified by elevated levels of monocytes and TH17 cells (Jodeleit et al., 2020a). Furthermore, we have shown that inflammation in UC is accompanied by elevated autoantibody levels, suggesting a breach of tolerance (Jodeleit et al., 2020b).

To translate these *ex vivo* observations into preclinical *in vivo* studies, we have developed a mouse model of UC, which is based on immune-compromised NOD/Scid IL2Rγ^null^ (NSG) mice reconstituted with peripheral blood mononuclear cells (PBMCs) from UC patients (NSG-UC). In this model, the immunological background of the donors is preserved (Jodeleit et al., 2020a). In the meantime, this model has been validated with various therapeutics, including infliximab, adalimumab, anti-CD1a antibodies and ritonavir (Jodeleit et al., 2017, 2020a; Palamides et al., 2016; Al-Amodi et al., 2018). Given that this model allows for testing of therapeutics directed against human target molecules, we tested the efficacy of oxelumab and compared it with the efficacy of adalimumab. The results suggest that oxelumab targets the pro-inflammatory condition while promoting the remodeling condition.

## RESULTS

### Expression of OX40 and OX40L in PBMCs of UC and non-UC donors

**Fig. 1.**
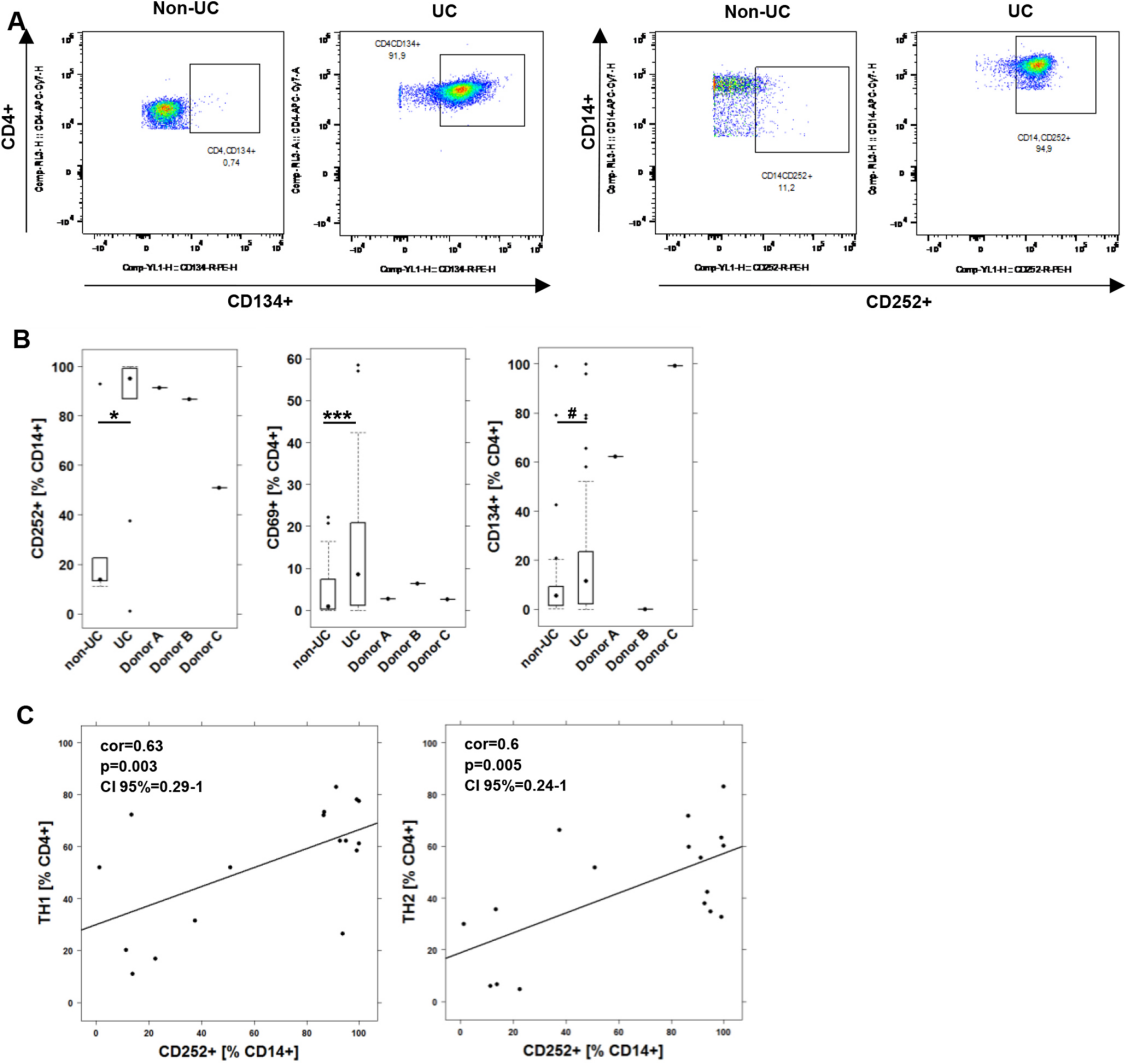
**Immune profiling of non-UC donors and UC patients.** (A) Representative flow cytometric images of CD4^+^ CD134^+^ and CD14^+^ CD252^+^ (OX40L) cells in PBMCs from non-UC and UC donors. (B) Frequencies of activated CD4^+^ T cells (CD134^+^ and CD69^+^) and CD252-expressing CD14^+^ monocytes depicted as boxplots. Boxes represent upper and lower quartiles; whiskers represent variability, and outliers are plotted as individual points. For comparison of groups, Student's unpaired *t*-test was conducted. Donor A-C refers to donors of PBMCs used in the animal study. ***P<0.001, *P<0.05, ^#^*P*<0.1. (C) Correlation analysis of OX40L (CD252) with TH1 and TH2 CD4^+^ T cells. The numbers display Pearson's product-moment correlation values (cor), *P*-values (*P*) and the 95% confidence interval (CI).

Given that OX40 and OX40L expression has been shown to increase in inflamed colons of UC patients (Souza et al., 1999), we analyzed PBMCs of UC patients (*n*=81) and non-UC donors (individuals with no apparent inflammation or metabolic aberrations; *n*=36) for frequencies of CD4^+^ CD134^+^ cells (for basic demographics, see Table 1). For comparison, the early activation marker CD69 was analyzed (for definition of cells by surface markers and gating strategy, see Table S2, Fig. S1). As shown in Fig. 1A,B, the frequencies of CD4^+^ CD134^+^ T cells were increased, but the differences just failed to be significant [non-UC, 12.11±21.27, mean±s.d.; UC, 21.54±25.71; *P*=0.054; 95% confidence interval (CI): −19.06 to 0.17]. By contrast, frequencies of CD4^+^ T cells expressing the early activation marker CD69 were significantly induced in UC patients (non-UC, 4.24±6.43; UC, 12.98±14.46; *P*=8×10^−5^; 95% CI: −12.96 to −4.51). A significant difference was also observed for OX40L-expressing monocytes (non-UC, *n*=5, 30.7±34.92; UC, *n*=12, 85.47±21.17; *P*=0.02; 95% CI: −97.19 to −12.29). To find evidence for which cells might be affected by OX40L-expressing monocytes, Pearson's product-moment correlation analysis was performed (Fig. 1C). This analysis revealed that OX40L-expressing monocytes were correlated positively with TH1 cells [Pearson's product-moment correlation value (cor)=0.63; *P*=0.005; 95% CI: 0.20 to 1] and TH2 cells (cor=0.6; *P*=0.003; 95% CI: 0.29 to 1). This result suggests that the activation of CD14^+^ monocytes by CD252 might be a marker for the acute inflammatory condition that was suggested by Föhlinger et al. (2016).

**Table 1.**
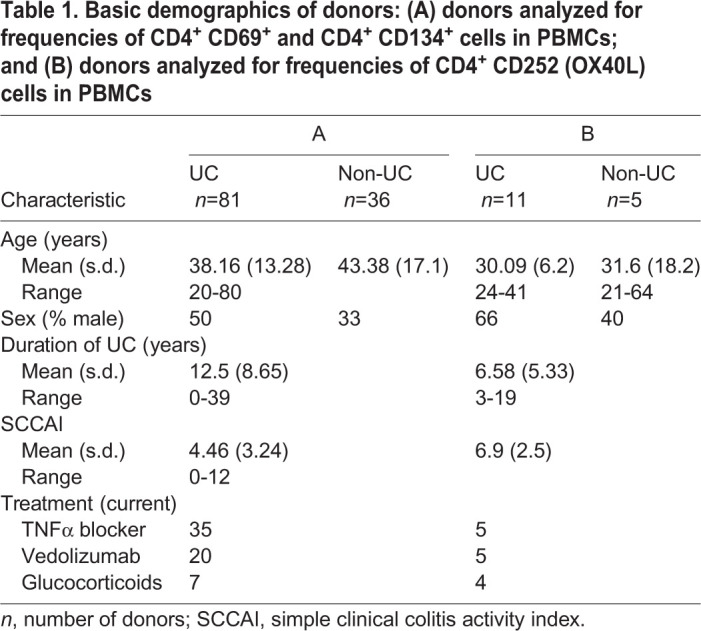
**Basic demographics of donors: (A) donors analyzed for frequencies of CD4^+^ CD69^+^ and CD4^+^ CD134^+^ cells in PBMCs; and (B) donors analyzed for frequencies of CD4^+^ CD252 (OX40L) cells in PBMCs**

### *In vivo* efficacy of oxelumab and adalimumab

**Fig. 2.**
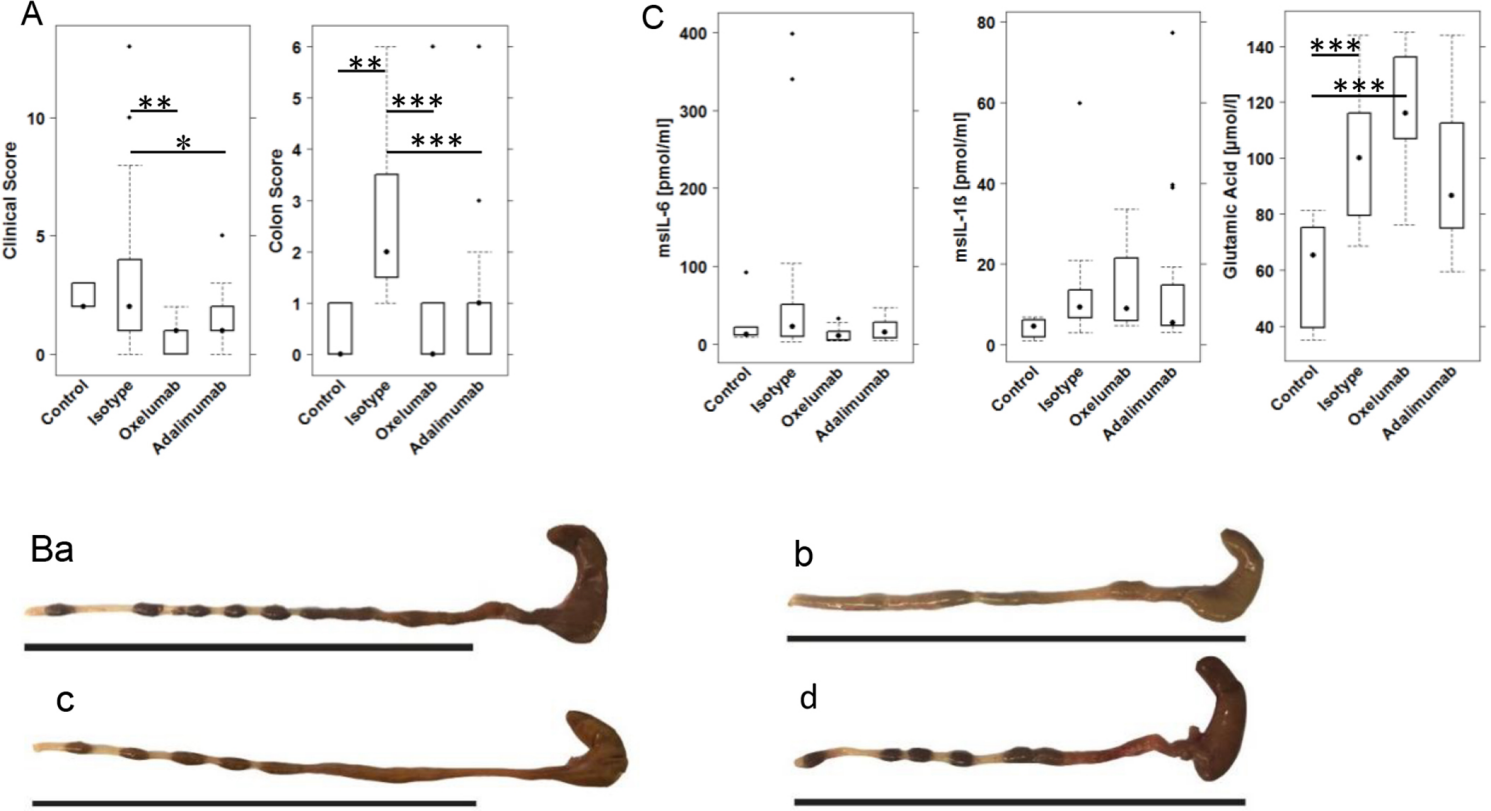
**Comparison of the efficacy of oxelumab and adalimumab *in vivo*.** NSG mice were engrafted with PBMCs derived from UC patients, challenged with 10% ethanol on day 7 and 50% ethanol on day 14 and treated with isotype (30 mg/kg), oxelumab (5 mg/kg) or adalimumab (30 mg/kg) on days 6 and 13: (a) unchallenged control (Control, *n*=6); (b) ethanol-challenged control treated with isotype (Isotype, *n*=24); (c) ethanol-challenged group treated with oxelumab (Oxelumab, *n*=12); and (d) ethanol-challenged group treated with adalimumab (Adalimumab, *n*=24). (A) Clinical and histological scores depicted as boxplots. (B) Representative photographs of colons at post-mortem examination: (Ba) unchallenged control; (Bb) challenged control treated with isotype; (Bc) challenged and treated with oxelumab; and (Bd) challenged and treated with adalimumab. (C) Mouse serum (ms) concentrations of IL-1β, IL-6 and glutamic acid depicted as boxplots. Boxes represent upper and lower quartiles; whiskers represent variability, and outliers are plotted as individual points. For comparison of groups, ANOVA followed by Tukey's HSD was conducted (***P<0.001, **P<0.01, *P<0.05).

NSG mice were reconstituted with PBMCs from UC patients and challenged according to a standard protocol (see Materials and Methods). Donors exhibited a simple clinical colitis activity index (SCCAI) (Walmsley et al., 1998) from six to nine and were treated with infliximab, corticoids and mezalazine or with azothioprine, mesalazine and glucocorticoids. As shown in Fig. 1B, all donors exhibited elevated levels of OX40L-expressing monocytes, whereas OX40 was elevated in only two donors. Eight days after reconstitution, the mice were divided into four groups, as shown in Table 2. On day 7, mice were challenged rectally with 10% ethanol, followed by challenge with 50% ethanol on day 14. Isotype (30 mg/kg), oxelumab (5 mg/kg) and adalimumab (30 mg/kg) were applied intraperitoneally on days 6 and 13 in 150 µl PBS. Upon challenge with ethanol, stools of the mice treated with isotype became soft or liquid, and the animals lost weight. Symptoms were classified according to a clinical score described in the Materials and Methods and indicated a slight increase in the isotype-treated group (3.11±3.25, mean±s.d.) compared with the control group (2.33±0.51; Fig. 2A), although the difference was not significant. The difference was significant, however, when the oxelumab-treated (0.94±0.72; *P*=0.006) or adalimumab-treated (1.45±1.14; *P*=0.04) mice were compared with the isotype-treated mice (for complete data set, see Table S3). Macroscopic inspection of the colons corroborated the clinical scores. As shown in Fig. 2B, the control colon of a mouse that was not challenged with ethanol appeared healthy, whereas the colon from the challenged and isotype-treated mouse was dilated and had lost the distinct pattern of pellets. Treatment with oxelumab or adalimumab reversed the appearance to normal. Colons were classified according to a colon score described in the Materials and Methods. As shown in Fig. 2A, the colon score increased significantly when control mice (0.33±0.51) were compared with isotype-treated mice (2.75±1.7; *P*=0.002) and decreased significantly in the oxelumab-treated (0.55±1.42; *P*=4×10^−5^ versus isotype) and adalimumab-treated (0.87±1.32; *P*=1×10^−4^ versus isotype) groups. The difference between both these groups and the control group was not significant.

**Table 2.**
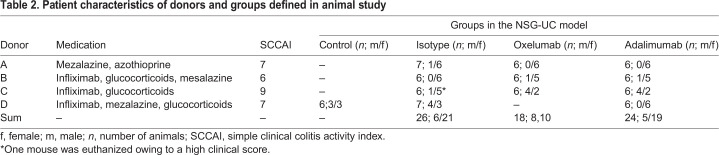
**Patient characteristics of donors and groups defined in animal study**

Before post-mortem examination, serum samples were collected and subjected to cytokine and amino acid analysis. As shown in Fig. 2C, concentrations of cytokines considered as inflammatory markers, such as IL-1β [control, 4.23±2.41; isotype, 12.53±12.2; *P*=not significant (n.s.)] and IL-6 (control, 26.29±32.32; isotype, 56.51±99.83; *P*=n.s.), increased in response to challenge, and IL-6 decreased upon treatment with oxelumab (12.49±8.89). No effect was observed on IL-1β and IFNγ. Concentrations of IL-4 increased in response to treatment with oxelumab or adalimumab (Table S3), suggesting that the wound-healing arm of inflammation was not suppressed and might even be promoted. Given that glutamic acid has recently been identified as an inflammatory marker in the NSG-UC mouse model (Jodeleit et al., 2018), concentrations of glutamic acid were determined in all groups. As shown in Fig. 2C, concentrations of glutamic acid increased significantly in response to challenge with ethanol (control, 60.25±19.46; isotype, 100.79±21.13; *P*=0.001) and decreased significantly in the adalimumab-treated group when compared with isotype (91.61±24.36; *P*=0.004). In the oxelumab-treated group, concentrations increased further (117.96±19.65), suggesting that this antibody promoted some form of inflammation.

**Fig. 3.**
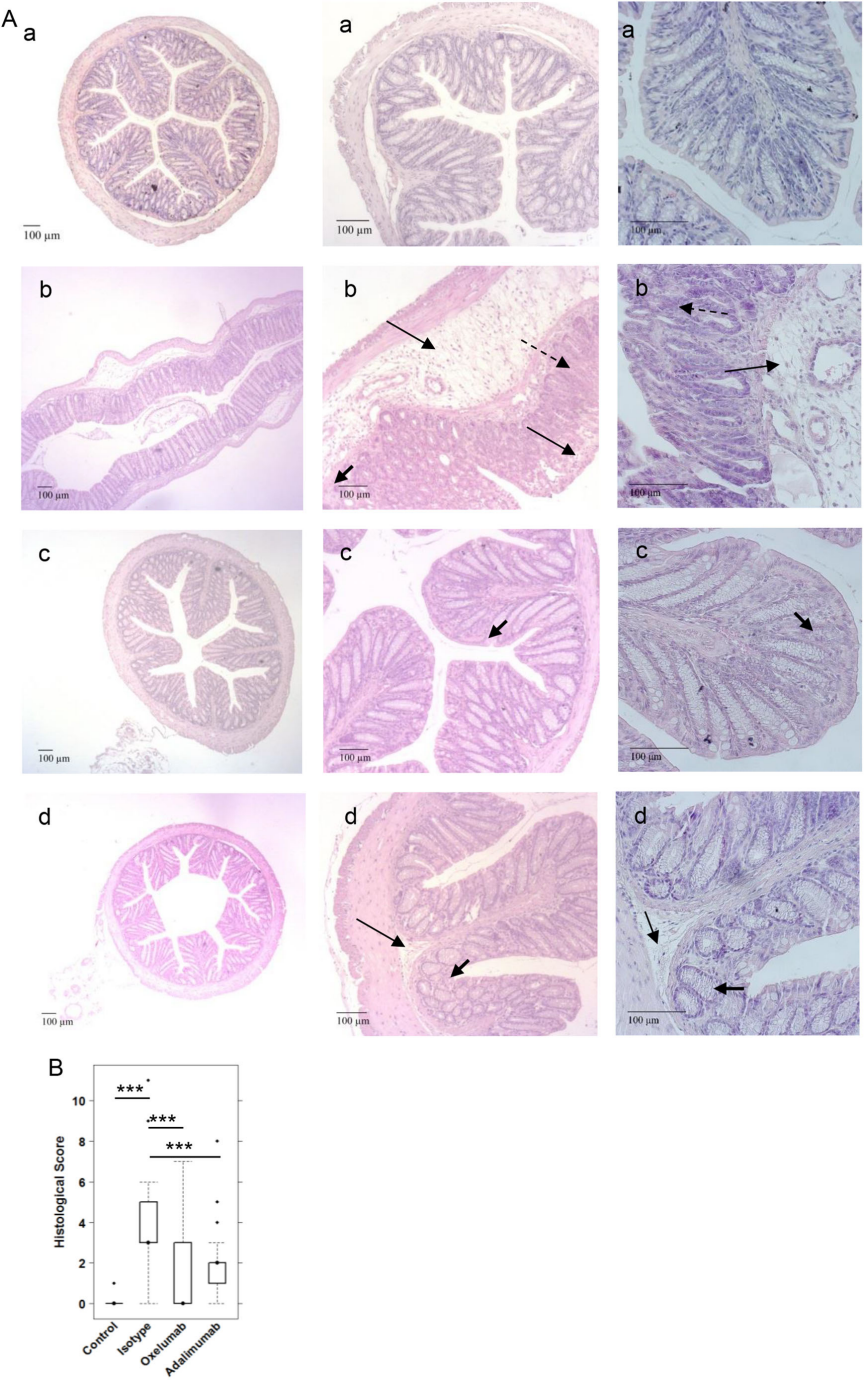
**Comparison of the efficacy of oxelumab and adalimumab *in vivo*.** Mice were treated as described in Fig. 2. (A) Representative photomicrographs of H&E-stained sections of distal parts of the colon from mice: (Aa) unchallenged control; (Ab) ethanol challenged control treated with isotype; (Ac) ethanol challenged group treated with oxelumab; and (Ad) ethanol challenged group treated with adalimumab. Arrows indicate edema and influx of inflammatory cells; dashed line arrows indicate destruction of crypts; bold arrows indicate fibrosis. (B) Histological scores depicted as boxplots. Boxes represent upper and lower quartiles; whiskers represent variability, and outliers are plotted as individual points. For comparison of groups, ANOVA followed by Tukey's HSD was conducted (***P<0.001).

As in previous studies (Jodeleit et al., 2017; Palamides et al., 2016; Al-Amodi et al., 2018), histological analysis of the colon revealed the influx of a mixed infiltrate of leukocytes, edema, crypt loss, and changes in the colonic mucosal architecture (Fig. 3A). To visualize fibrosis, sections were also stained with Elastica van Gieson (Fig. S2). The histopathological manifestations were classified according to a histological score (Fig. 3B; Table S4) and confirmed a significant response to ethanol in the isotype-treated group (4.08±2.3) compared with the control group (0.166±0.40; *P*=1×10^−4^) and the ameliorating effect of oxelumab (1.58±2.3; *P*=0.001 versus isotype) and adalimumab (2.08±1.69, *P*=0.005) versus isotype.

**Fig. 4.**
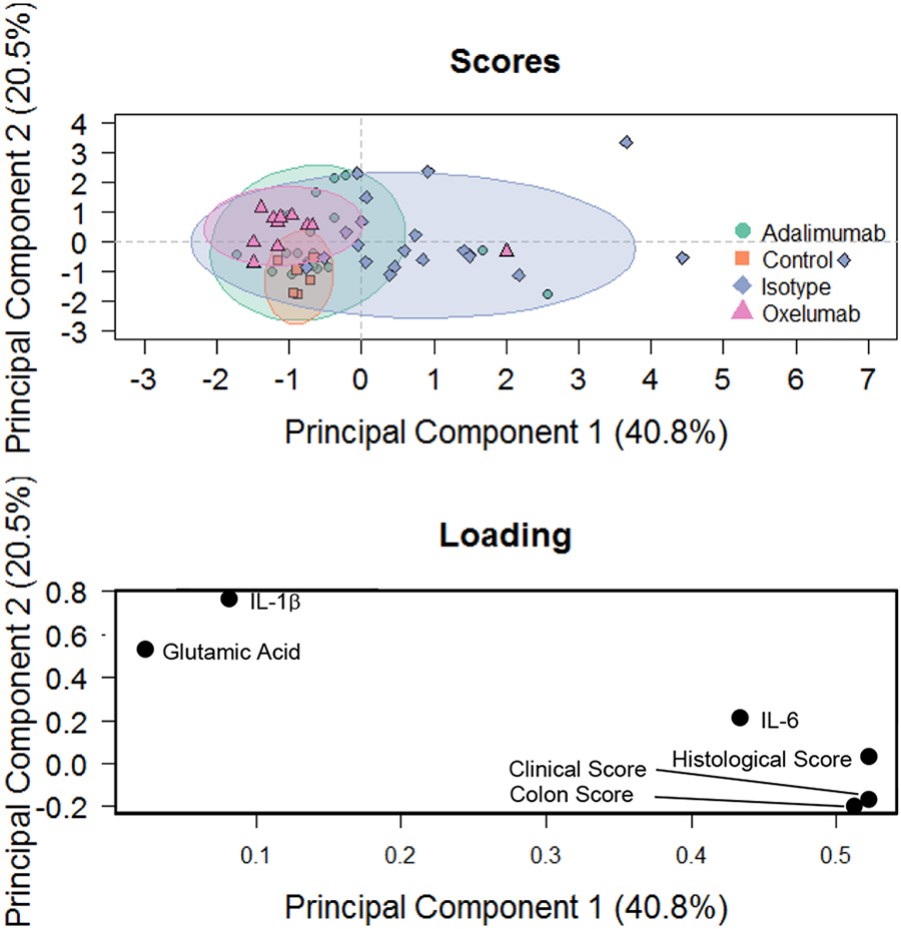
**Principal components analysis.** Mice were treated as described in Fig. 2. Clinical, colon and histological scores and serum concentrations of IL-6, IL-1β and glutamic acid were used as variables.

**Fig. 5.**
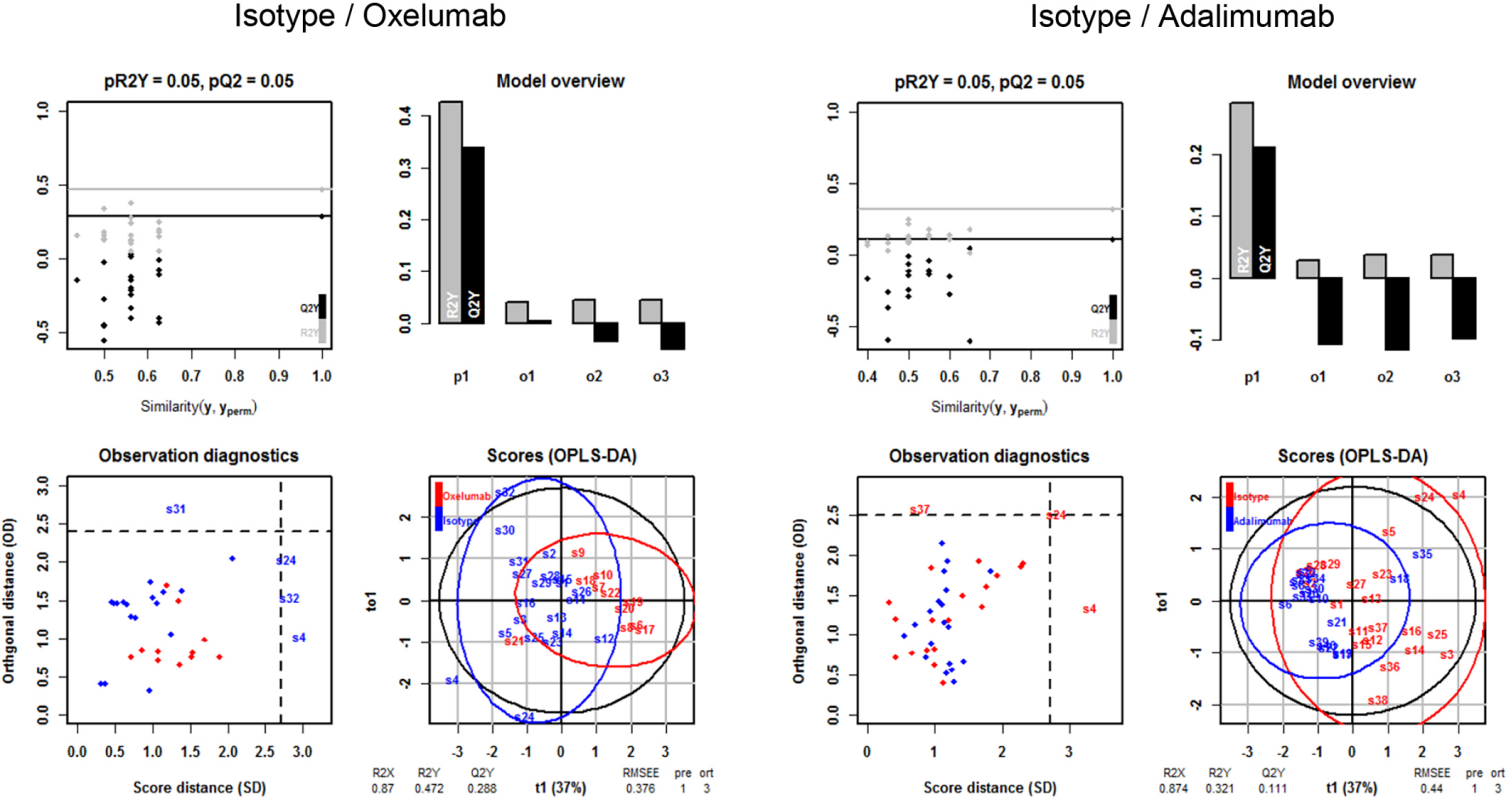
**Comparison of the efficacy of oxelumab and adalimumab *in vivo* by orthogonal partial least squares discrimination analysis.** NSG-UC mice were treated as described in Fig. 2. Variables used were clinical, colon and histological scores and serum concentrations of IL-6, IL-1β and glutamic acid. Q^2^Y, fraction of variation of the y variables predicted by the model; R^2^X, fraction of the variation of the X variables explained by the model; R^2^Y, fraction of the variation of the Y variables explained by the model; RMSEE, root mean square error of estimation.

In order to evaluate the efficacy of treatment, a principal components analysis (PCA) was performed, including the clinical, colon and histological scores and the concentrations of IL-6, IL-1β and glutamic acid as variables. As shown in Fig. 4, samples of the control group clustered closely together. Upon challenge with ethanol, samples spread out, reflecting the variability seen in this model. In response to oxelumab and adalimumab, samples again clustered more closely but were still distinct from the control group. This analysis indicated that oxelumab exhibited similar efficacy to adalimumab. In order to quantify the difference between the oxelumab and adalimumab groups, an orthogonal partial least squares discriminating analysis (oPLS-DA) was performed. As shown in Fig. 5, the oxelumab- and adalimumab-treated mice displayed some overlap with the isotype-treated mice, but the discrimination was significant as indicated by the pQ^2^ (quality assessment) value of 0.05. Oxelumab seemed to be slightly more efficacious than adalimumab, as indicated by the higher R^2^Y (fraction of variation of Y variables explained by the model value) value of 0.288 versus 0.111 and the lower root mean square error of estimation (RMSEE) value of 0.37 in the oxelumab versus isotype analysis, compared with 0.44 in the adalimumab versus isotype analysis. These results indicate that oxelumab is more effective than adalimumab; however, mice did not achieve the inflammatory status of the control mice.

**Fig. 6.**
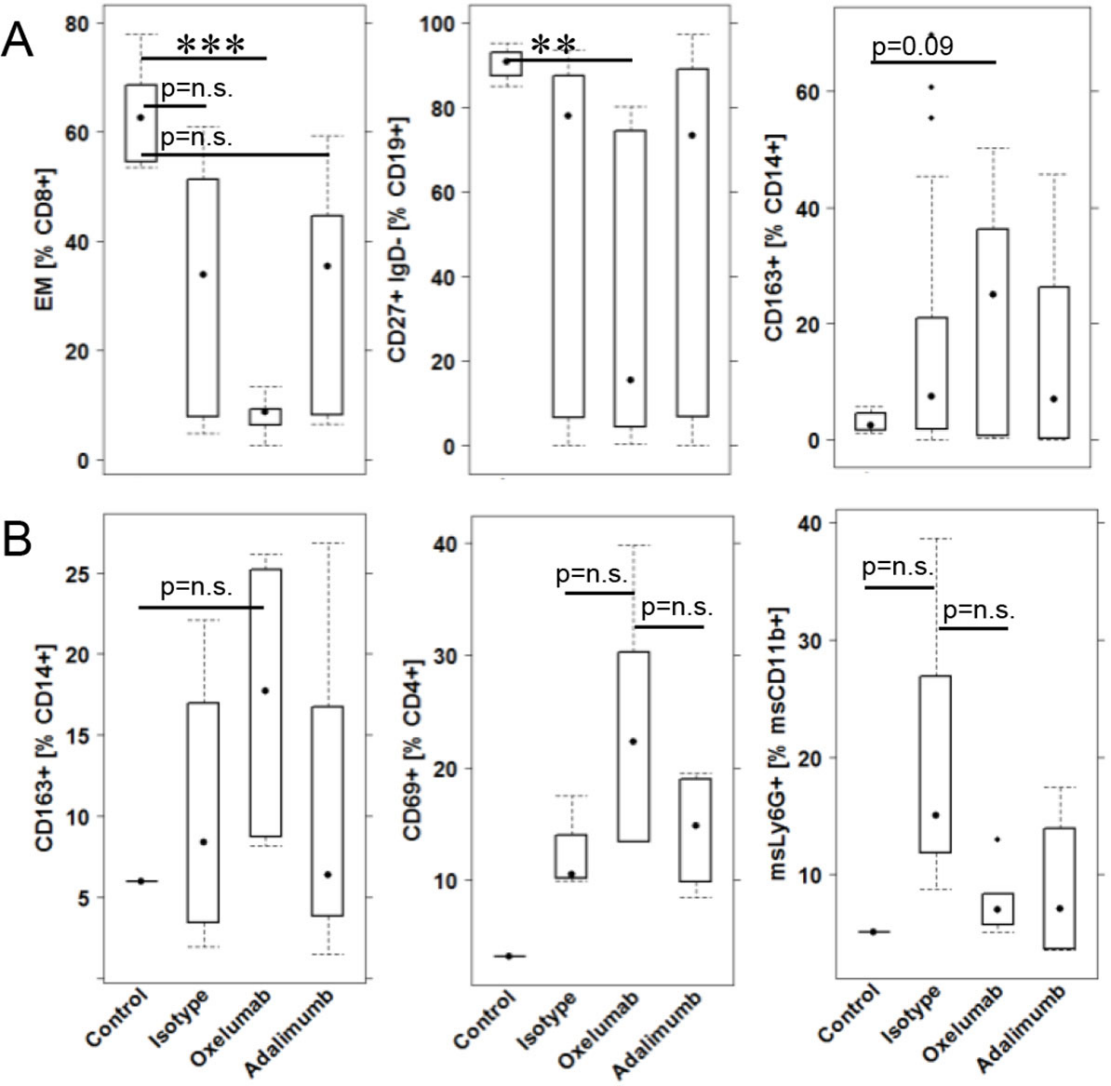
**Impact of treatment with oxelumab and adalimumab on leukocytes.** (A) Splenic leukocytes. (B) Colonic leukocytes. Mice were treated as described in Fig. 2. Boxes represent upper and lower quartiles; whiskers represent variability, and outliers are plotted as individual points. For comparison of groups, ANOVA followed by Tukey's HSD was conducted. Labels given on the *x*-axes on the bottom row apply to all charts. For comparison of groups, ANOVA followed by Tukey's HSD was conducted (***P<0.001, **P<0.01; n.s., not significant).

To analyze the effect of oxelumab and adalimumab on cell types, leukocytes were isolated from spleens and colons of mice and subjected to flow cytometric analysis (for use of markers and gating strategy, see Fig. S1; Table S2). As shown in Fig. 6 and Table S3, no significant effect of oxelumab on splenic leukocytes was observed, with the exception of CD8^+^ effector memory T cells. Here, a significant decline was detected when frequencies of the control group (63.31±29.32) were compared with those of the oxelumab-treated group (8.47±2.31; *P*=0.003).

No significant impact of treatment with oxelumab on colonic leukocytes was observed. However, a trend was observed that indicated a general impairment of active inflammation by oxelumab and adalizumab, shown by a decrease of mouse neutrophils. In addition, the increase of M2 monocytes (CD14^+^ CD163^+^; *P*=n.s.) and activated CD4^+^ cells (CD4^+^ CD69^+^; *P*=n.s.) induced by oxelumab might suggest ongoing wound-healing processes. This might also explain the elevated glutamic acid concentrations.

## DISCUSSION

### Patient profiling

Treatment decisions for UC often rely on an escalating strategy, with trial and error. Therefore, a major goal is to stratify patients for therapies to avoid unnecessary treatments, with their accompanying side effects, in the absence of clinical benefit. Given that UC is an umbrella diagnosis covering multiple disease forms distinguished by their manifestation, severity, course and response to therapeutics, this goal remains highly ambitious. Traditionally, T cell-mediated inflammation has been the focus of research in inflammatory bowel disease, according to the hypothesis that UC was a TH2 cell-driven disease, whereas in Crohn's disease (CD) the inflammation was characterized by TH1 cells (Heller et al., 2002, 2005; Brand, 2009). Based on this assumption, anti-TNFα (also known as TNF) antibodies and ustekinumab were first developed for CD and only later approved or tested for UC (Sands et al., 2019). This view, however, neglected the role of epithelial cells and monocytes in inducing and shaping inflammation and the dynamics of inflammatory processes (Pastorelli et al., 2013; Liu et al., 2007; Foucher et al., 2013) to include protective and healing functions. This view also disregarded the impact of intestinal stromal cells, which also have the capacity to promote inflammation in a T cell- and TNFα-independent inflammation mediated by oncostatin M (West et al., 2017; Beigel et al., 2014). Of note, oncostatin M is now considered a significant biomarker for anti-TNFα-resistant inflammation. Furthermore, the impact of inflammation and therapeutics on metabolism has become a focus of interest (Dawiskiba et al., 2014; Bjerrum et al., 2017; Biemans et al., 2020; Miranda-Bautista et al., 2015). Inflammation itself is thought to induce a switch to glycolysis, and therapeutics such as tofacitinib and infliximab cause increased cholesterol levels. Therefore, metabolic effects have to be considered in preclinical development.

It is a prerequisite for personalized and phase-dependent therapies to stratify patients according to their individual immunological profile. In a previous study, we were able to show that immune profiling of patients distinguished subgroups of patients (Jodeleit et al., 2020a). One group was characterized predominantly by elevated levels of TH1 and TH2 cells, whereas the other group was signified by elevated levels of M1 monocytes (CD14^+^ CD64^+^), suggesting at least two inflammatory conditions: pro-inflammatory and remodeling. Longitudinal studies have shown that these subgroups reflect the dynamics of the inflammatory processes, as patient profiles switched from one group to the other. This previous analysis failed to correlate OX40-expressing CD4^+^ T cells with both groups. In the present study, we showed that the early CD4^+^ T cell activation marker (CD69^+^) was significantly elevated in UC patients compared with non-UC donors. By contrast, the difference between the two groups was not as clear when the late activation marker (OX40) was analyzed, suggesting that expression might relate to subgroups of patients. Therefore, we could not predict a potential response to oxelumab from this analysis. In addition to OX40 expression, monocytes expressing OX40L (CD252) were analyzed in a smaller study group. Here, we observed a significant difference between non-UC and UC donors. Frequencies of CD14^+^ CD252^+^ cells were correlated significantly with TH1 and TH2 cells, suggesting that activated monocytes might have an impact on TH1 and TH2 cells.

### *In vivo* efficacy of oxelumab

To date, the translation of *ex vivo* data obtained from patients into a preclinical *in vivo* animal model presents a major challenge. To narrow this gap, we combined immune profiling of patients with an animal model that partly reflects the immune status of the donor and allows for the testing of therapeutics directed against human target molecules. In the NSG-UC model, mice benefited from treatment with oxelumab, as indicated by significantly reduced clinical, colon and histological scores and reduced serum concentrations of IL-6. However, the metabolic marker glutamic acid was not reduced but significantly elevated compared with the isotype group, suggesting ongoing inflammatory processes. This observation was corroborated by IL-1β and glutamic acid concentrations, which did not decrease upon treatment with oxelumab. Treatment with oxelumab caused a significant decline in splenic effector CD8^+^ T cells and switched B cells, suggesting that oxelumab affected the generation of these cells and thereby restored tolerance. By contrast, frequencies of M2 monoytes (CD14^+^ CD163^+^) increased, albeit not significantly, confirming an ongoing inflammatory process. The analysis of colonic leukocytes also corroborated this observation. Frequencies of M2 monocytes and activated CD4^+^ T cells increased further upon treatment with oxelumab. The observation that frequencies of colonic mouse neutrophils decreased in response to treatment confirmed that oxelumab addressed the pro-inflammatory condition and that mice benefited from treatment with oxelumab. The fact that M2 monocytes were not affected indicated that oxelumab had an impact on the pro-inflammatory process but not the remodeling process. The observed increase in concentrations of glutamic acid also suggested ongoing inflammation.

The study presented here corroborates the findings of previous *in vivo* studies that validated OX40L as a therapeutic target (Obermeier et al., 2003; Totsuka et al., 2003; Malmström et al., 2001). In the study by Totsuka et al. (2003), the efficacy of oxelumab was tested in a mouse model of UC that relies on adoptive transfer into SCID mice of CD4^+^ CD45RB^high^ cells isolated from BALB/c mice. Here, the histological score was reduced by ∼50%. This observation led to a combinatorial study with anti-TNFα antibodies, which showed further amelioration of inflammation. In the meantime, humanised mouse models have been improved to allow for reliable reconstitution of PBMCs (King et al., 2007). The major advantage of the NSG-UC mouse model lies in the combination of patient profiling and testing with selected donors. The present study suggests that like adalimumab, oxelumab addresses the pro-inflammatory arm of inflammation with high efficacy. Therefore, we expect that the combination with anti-TNFα would not ameliorate the pathological phenotype further. However, it would be an intriguing idea to examine whether mice reconstituted with PBMCs from patients resistant to anti-TNFα treatment would benefit from treatment with oxelumab. Alternatively, a combined treatment with a therapeutic simultaneously addressing the remodeling arm of inflammation could ameliorate inflammation further.

## MATERIALS AND METHODS

### Ethical considerations

Written, informed consent was given by all donors. The study was approved by the Institutional Review Board (IRB) of the Medical Faculty at the University of Munich (120-15).

Animal studies were approved by the animal welfare committees of the government of Upper Bavaria, Germany (55.2-2-1-54-2532-74-15) and performed in compliance with German Animal Welfare Laws.

### Isolation of PBMCs and engraftment

Sixty milliliters of peripheral blood was collected into trisodium citrate solution (S-Monovette; Sarstedt, Nürnberg, Germany) from the arm vein of UC patients. The blood was diluted with Hank's balanced salt solution (HBSS; Sigma-Aldrich, Deisenhofen, Germany) in a 1:2 ratio. The suspension was loaded into LeucoSep tubes (Greiner Bio One, Frickenhausen, Germany). The PBMCs were separated by centrifugation at 400 ***g*** for 30 min and no acceleration. The interphase was extracted and diluted with PBS to a final volume of 40 ml. Cells were counted and centrifuged at 1400 ***g*** for 5 min. The cell pellet was resuspended in PBS at a concentration of 4×10^6^ cells in 100 µl.

Six- to 8-week-old NOD.cg-Prkdc^SCID^ Il2rg^tm1Wjl^/Szj mice (abbreviated as NOD/Scid IL2Rγ^null^; NSG) were engrafted with 100 µl cell solution into the tail vein on day 1.

### Study protocol

NSG mice were obtained from Charles River Laboratories (Sulzfeld, Germany). The study followed the protocol described in a previous study (Palamides et al., 2016). Mice were kept in specific pathogen-free conditions in individually ventilated cages in a facility controlled according to the Federation of Laboratory Animal Science Association (FELASA) guidelines. After engraftment on day 0, mice were presensitized by rectal application of 150 µl of 10% ethanol on day 7 using a 1 mm cat catheter (Henry Schein, Hamburg, Germany). The catheter was lubricated with Xylocain Gel 2% (AstraZeneca, Wedel, Germany). Rectal application was performed under general anesthesia using 4% isoflurane (Zoetis, Berlin, Germany). After application, mice were kept at an angle of 30° to avoid ethanol dripping. On day 14, mice were challenged by rectal application of 50% ethanol, following the protocol of day 7. On day 18, mice were sacrificed. Adalimumab and oxelumab were provided by Sanofi-Aventis Deutschland (Frankfurt am Main, Germany) and were applied in PBS (30 mg/kg/day or 5 mg/kg/day, respectively) on days 6 and 13. The control groups were injected with 30 mg/kg of the isotype antibody (Sanofi-Aventis Deutschland) on days 6 and 13. Dosage of oxelumab was according to a clinical phase II study proving the efficacy in asthma (Gauvreau et al., 2014), and dosage of adalimumab followed the dosage recommendation for children (https://www.rxlist.com/humira-drug.htm). Mice were sacrificed on day 18.

### Clinical activity score

The assessment of colitis severity was performed daily according to the following scoring system: loss of body weight: 0% (0), 0-5% (1), 5-10% (2), 10-15% (3), 15-20% (4); stool consistency: formed pellet (0), loose stool or unformed pellet (2), liquid stools (4); behavior: normal (0), reduced activity (1), apathy (4), ruffled fur (1); body posture: intermediately hunched posture (1), permanently hunched posture (2). The scores were added daily into a total score with a maximum of 12 points per day. Animals that suffered from weight loss of more than 20%, rectal bleeding, rectal prolapse, self-isolation or a severity score greater than seven were euthanized immediately and not assessed in the results. All scores were added for statistical analysis.

### Colon score

The colon was removed; a photograph was taken, and the colon was scored as follows: pellet: formed (0), soft (1), liquid (2); length of colon: >10 cm (0), 8-10 cm (1), <8 cm (2); dilation: no (0), minor (1), severe (2); hyperemia: no (0), yes (2); necrosis: no (0), yes (2).

### Histopathological analysis

At post-mortem examination, samples from distal parts of the colon were fixed in 4% formaldehyde for 24 h before storage in 70% ethanol and were routinely embedded in paraffin. Samples were cut into sections 3 µm in thickness and stained with Hematoxylin and Eosin (H&E). Epithelial erosions were scored as follows: no lesions (1), focal lesions (2), multifocal lesions (3), major damage with involvement of basal membrane (4). Inflammation was scored as follows: infiltration of few inflammatory cells into the lamina propria (1), major infiltration of inflammatory cells into the lamina propria (2), confluent infiltration of inflammatory cells into the lamina propria (3), infiltration of inflammatory cells including tunica muscularis (4). Fibrosis was scored as follows: focal fibrosis (1), multifocal fibrosis and crypt atrophy (2). The presence of edema, hyperemia and crypt abscess was scored with one additional point in each case. The scores for each criterion were added into a total score ranging from zero to 12. Images were obtained with an AxioVert 40 CFL camera (Zeiss, Oberkochen, Germany). The figures show representative longitudinal sections at the original magnification. In Adobe Photoshop CC, a tonal correction was used in order to enhance the contrast within the pictures.

### Isolation of human leukocytes

To isolate human leukocytes, mouse spleens were minced and cells filtered through a 70 µm cell strainer followed by centrifugation at 1400 ***g*** for 5 min and resuspension in FACS buffer [1×PBS, 2 mM EDTA and 2% fetal calf serum (FCS)]. For further purification, cell suspensions were filtered using a 35 µm cell strainer and labeled for flow cytometry analysis.

For isolation of lamina propria mononuclear cells (LPMCs) from colons of mice, a protocol modified from that of Weigmann et al. (2007) was used. The washed and minced colon was pre-digested in an orbital shaker with slow rotation (40 g) at 37°C for 20 min in pre-digesting solution containing 1× HBSS (Thermo Fisher Scientific, Darmstadt, Deutschland), 5 mM EDTA, 5% FCS and 100 U/ml pencillin-streptomycin (Sigma-Aldrich, St Louis, MO, USA). Epithelial cells were removed via filtration through a nylon filter. After washing with RPMI (Thermo Fisher Scientific), the remaining pieces of colon were digested for 2×20 min in digestion solution containing 1× RPMI, 10% FCS, 1 mg/ml collagenase A (Sigma-Aldrich), 10 KU/ml Dnase I (Sigma-Aldrich) and 100 U/ml pencillin-streptomycin (Sigma-Aldrich) in an orbital shaker with slow rotation (40 ***g***) at 37°C (Weigmann et al., 2007).

Isolated LPMCs were centrifuged at 1400 ***g*** for 5 min and resuspended in FACS buffer. Cell suspensions were filtered once more using a 35 µm cell strainer for further purification before the cells were labeled for flow cytometric analysis.

### Flow cytometric analysis

Labeling of human leukocytes was performed according to Table S1.

All antibodies were purchased from BioLegend (San Diego, CA, USA) and used according to the manufacturer's instructions. Flow cytometry was performed using a BD FACS Canto II and analysed with FlowJo v.10.1 software (FlowJo, Ashland, OR, USA).

### Detection of cytokines in sera and colon

Whole blood was collected and allowed to clot at room temperature for 30 min. After 10 min of centrifugation at 2000 ***g*** and 4°C, the supernatant was transferred to a fresh polypropylene tube and used immediately or stored at −80°C.

Sections of the terminal colon ∼10 mm in length were dissected and cleaned of feces with ice-cold PBS. Then, 500 µl protease inhibitor cocktail (cOmplete; Roche, Penzberg, Germany) was added according to the manufacturer's instructions. Samples were milled with a 5 mm stainless-steel bead (Qiagen, Hilden, Germany) and centrifuged for 5 min at 300 ***g***. Supernatants were shock-frozen and stored at −80°C.

Supernatants or sera were analysed for cytokine content either with the MSD Mesoscale platform (Meso Scale Diagnostics, Rockville, MD, USA) using the V-PLEX Proinflammatory Panel 1 Mouse Kit or with the LUNARIS platform (AYOXXA Biosystems, Cologne, Germany) for multiplex protein analysis using the LUNARIS Mouse 12-Plex Th17 or the Human 11-Plex Cytokine Kit, using the protocol provided by the respective manufacturers. Fluorescence signals of bound target proteins were recorded by high-resolution imaging for quantification by the proprietary LUNARIS Analysis Suite.

### Detection of amino acids

Serum samples were prepared according to the manufacturer's instructions. After incubation of 100 µl serum with internal standards for 5 min, 25 µl 15% 5-sulfosalicylic acid was added and samples were centrifuged at 9000 ***g*** for 15 min at 4°C. Supernatants were filtered through a 0.2 µm membrane, and 75 µl lithium loading buffer was added. Samples were analyzed using the amino acid analyzer Biochrom 30+ (Biochrom, Cambridge, UK).

### Statistical analysis

Statistical analysis was performed with R (https://www.R-project.org/). Variables were represented with mean, standard deviation and median values. Student's unpaired *t*-test and a 95% CI were used to compare binary groups, whereas for more than two groups, ANOVA followed by Tukey's HSD was conducted. Variables subjected to ANOVA were tested for normal distribution. All variables, with the exception of glutamic acid, fulfilled this requirement. For correlation analysis, Pearson's product-moment correlation was performed, and a 95% CI was applied. ANOVA followed by Tukey's HSD was conducted. For correlation analysis, Pearson's product-moment correlation was performed, and a 95% CI was applied. A heatmap was made using R (default). Principal components analysis (PCA) was performed using the plyr, ChemometricsWithR, maptools, car and rgeos packages. oPLS-DA was performed using the ropls package (Thévenot et al., 2015).

## Supplementary Material

Supplementary information
