# Supplementary material for: Head-to-head study of oxelumab and adalimumab in a mouse model of ulcerative colitis based on NOD/Scid IL2Rγnull mice reconstituted with human peripheral blood mononuclear cells
Source: Dis Model Mech. 2021 Jan 21;14(1):dmm046995. doi: 10.1242/dmm.046995 (PMC7847261; doi:10.1242/dmm.046995)
Supplement: Supplementary information [file dmm-14-046995-s1.pdf]

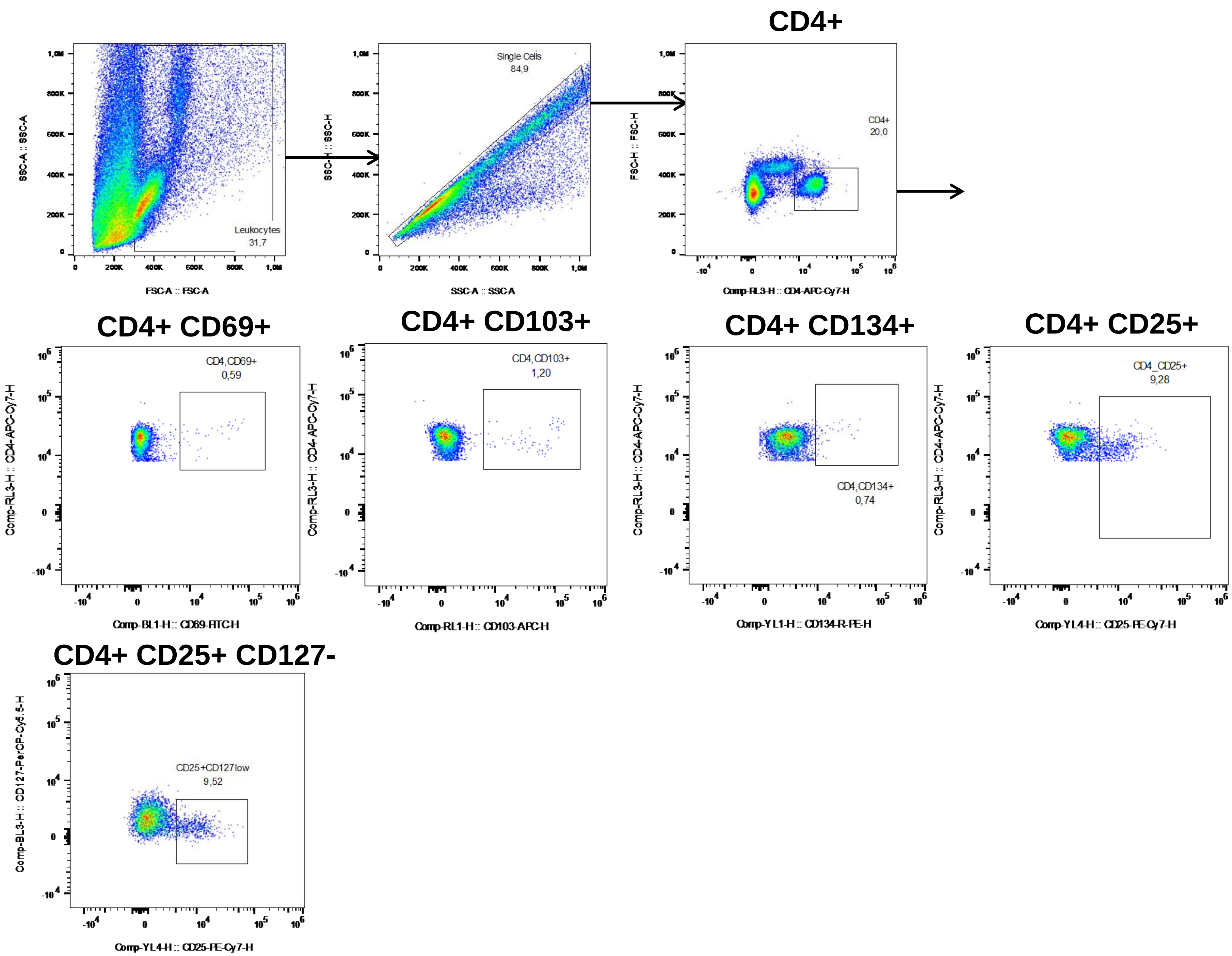

Disease Models & Mechanisms • Supplementary information

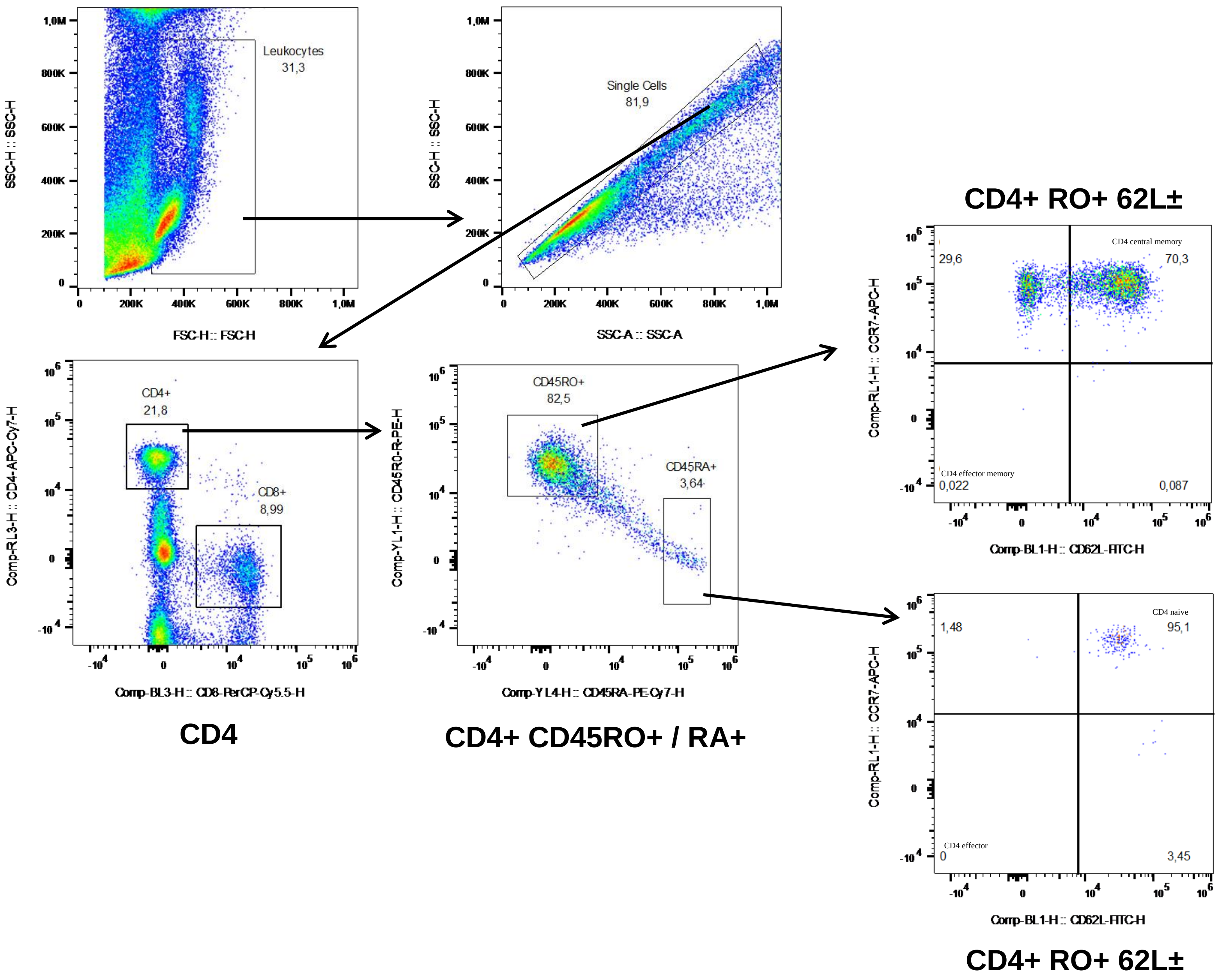

Disease Models & Mechanisms • Supplementary information

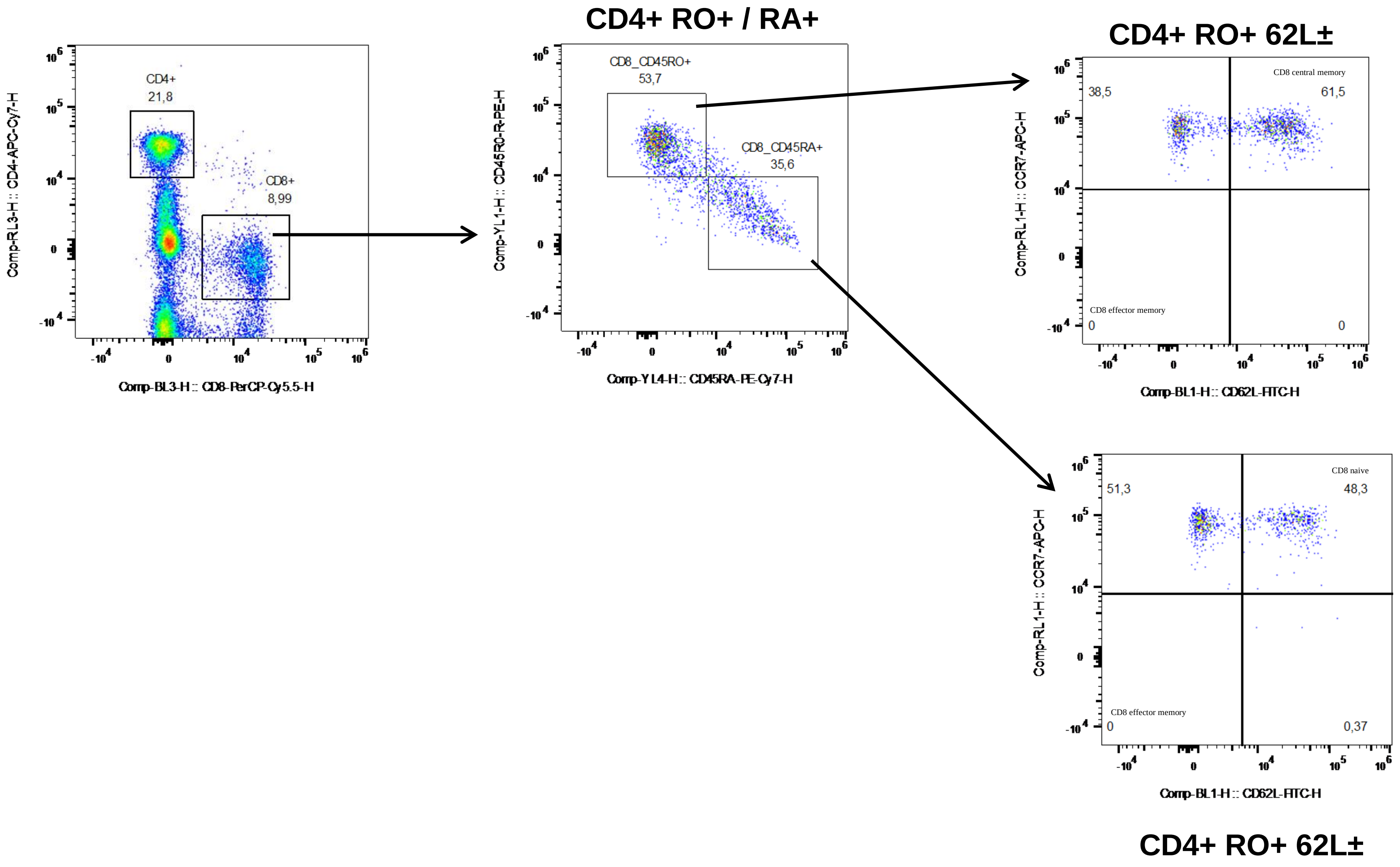

Disease Models & Mechanisms • Supplementary information

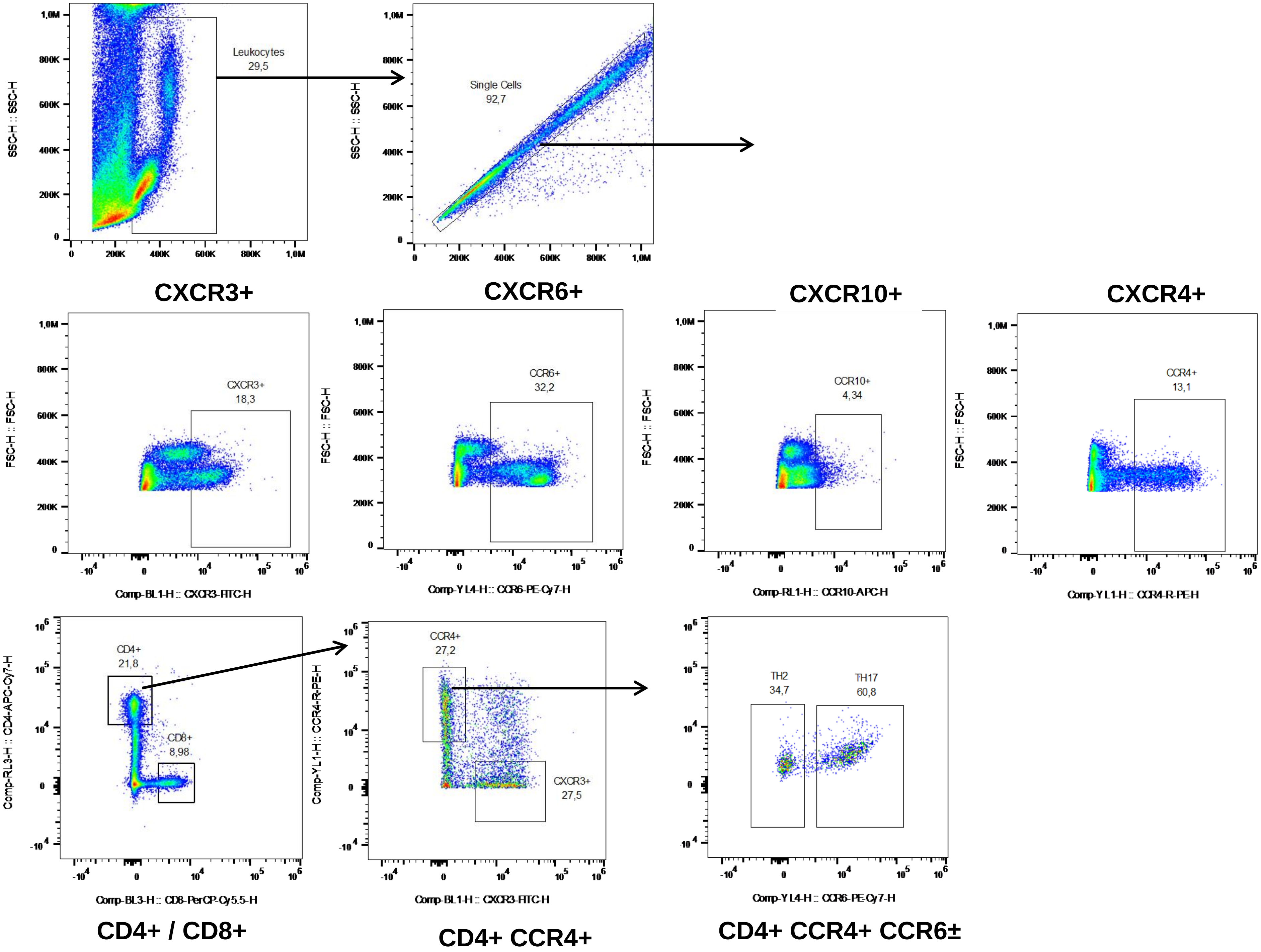

Disease Models & Mechanisms • Supplementary information

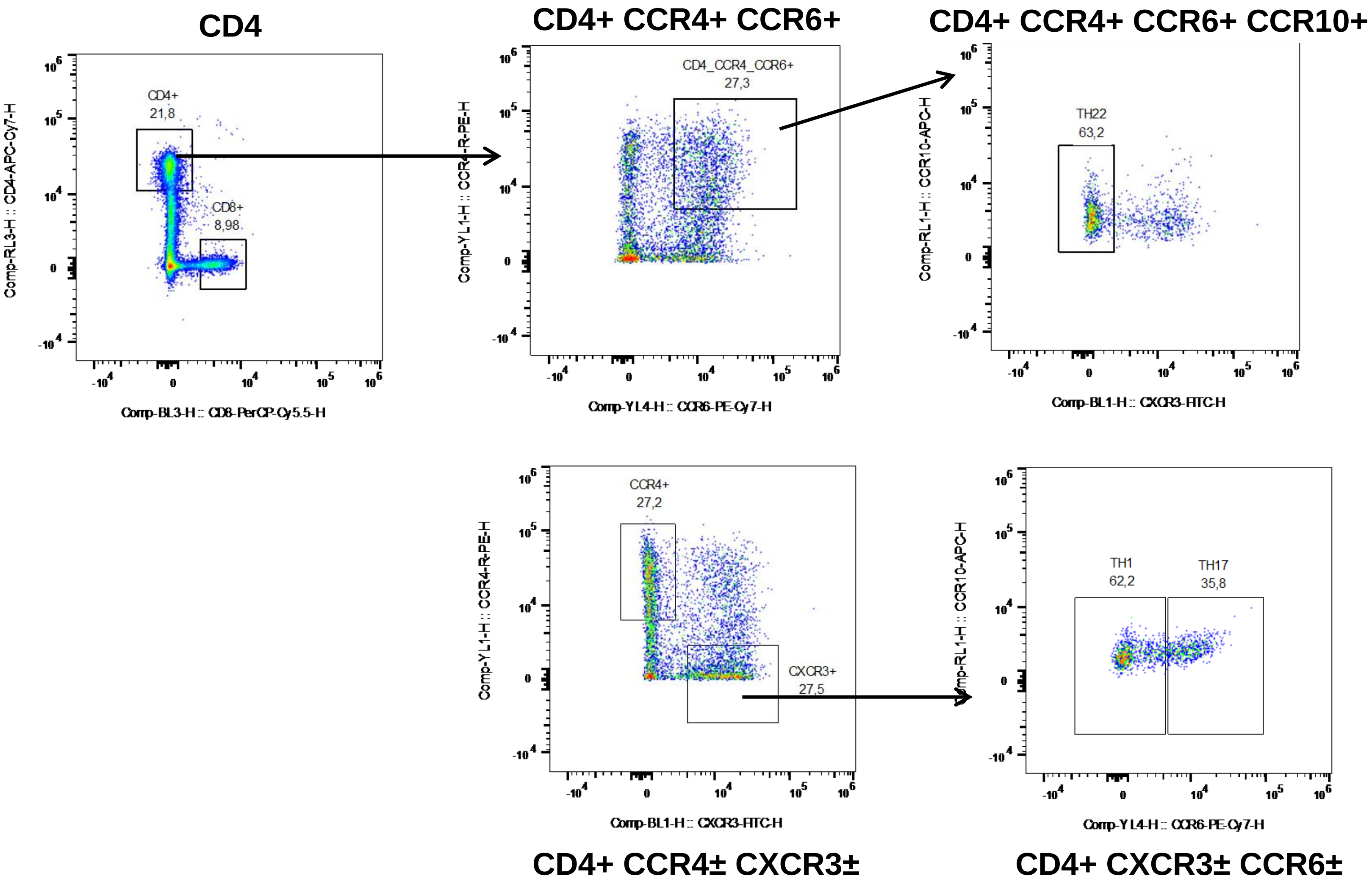

Disease Models & Mechanisms • Supplementary information

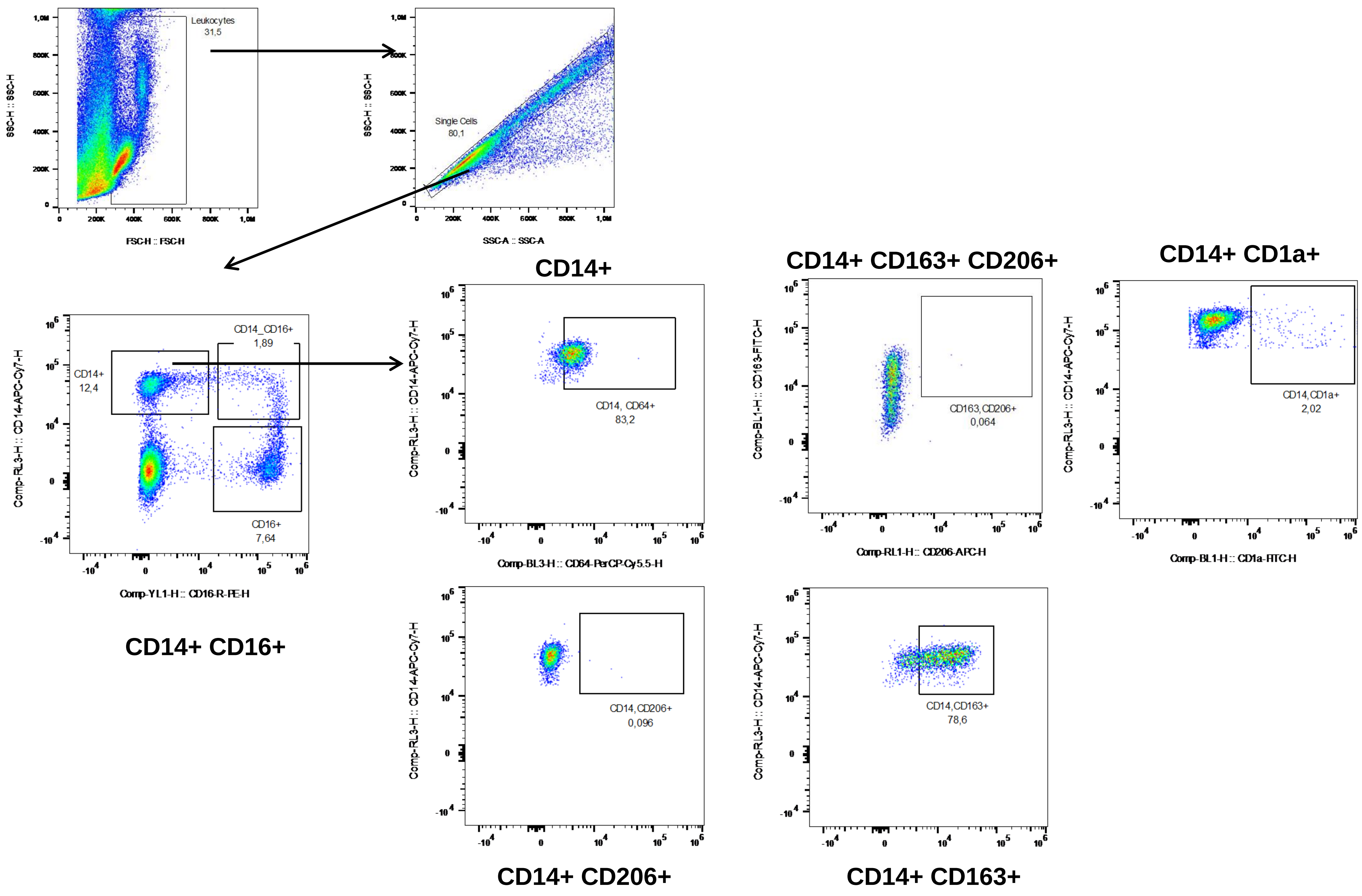

Disease Models & Mechanisms • Supplementary information

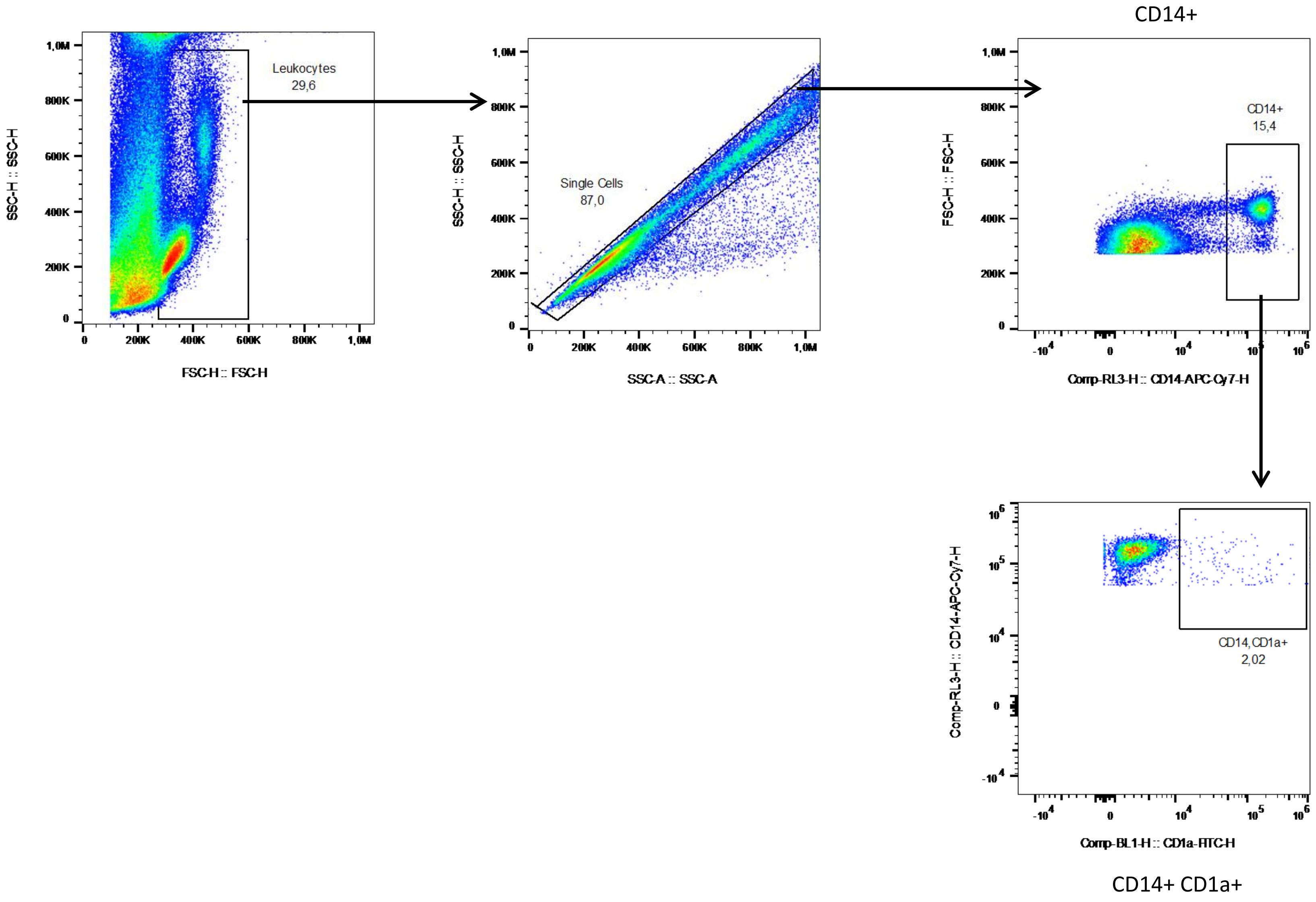

Disease Models & Mechanisms • Supplementary information

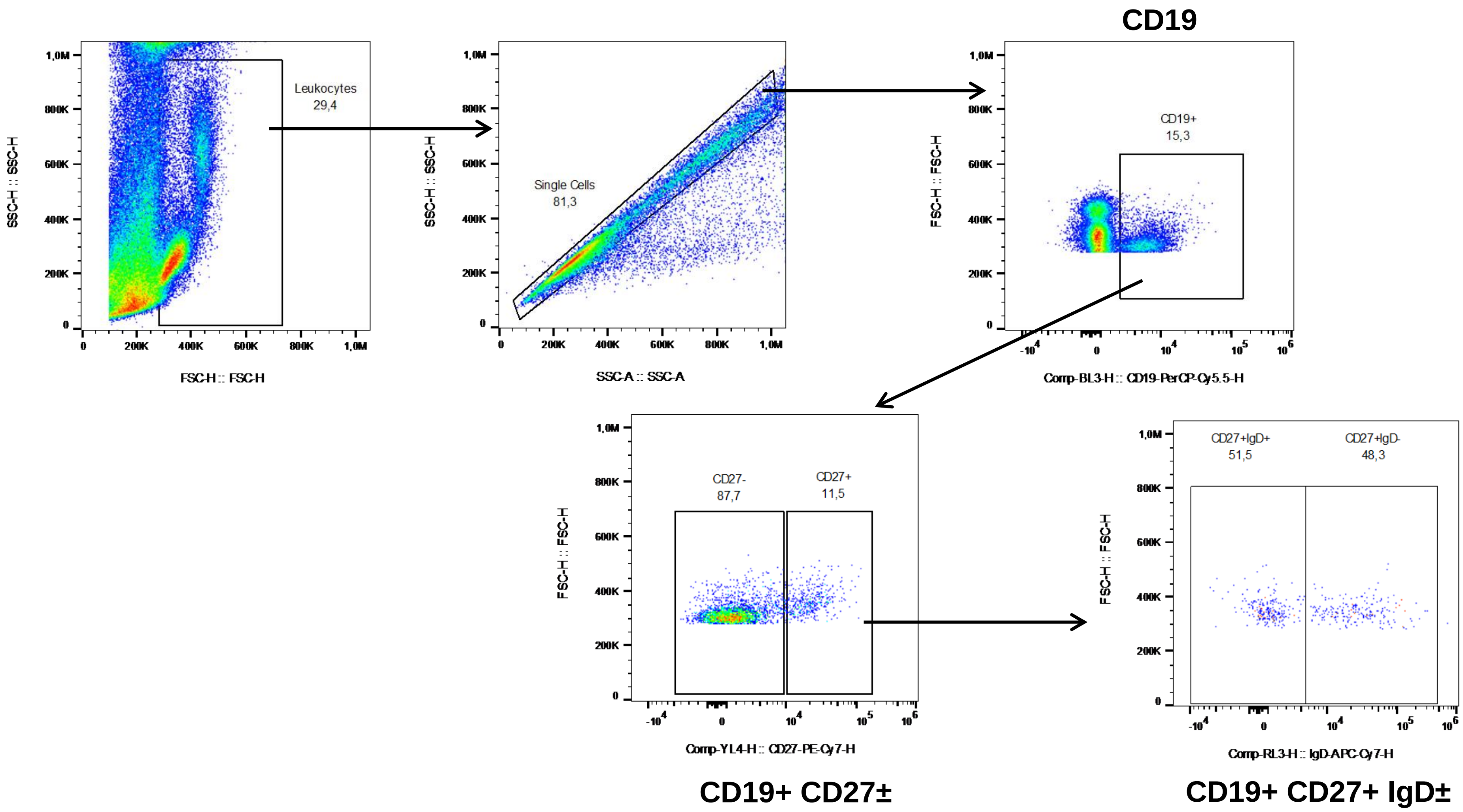

Disease Models & Mechanisms • Supplementary information

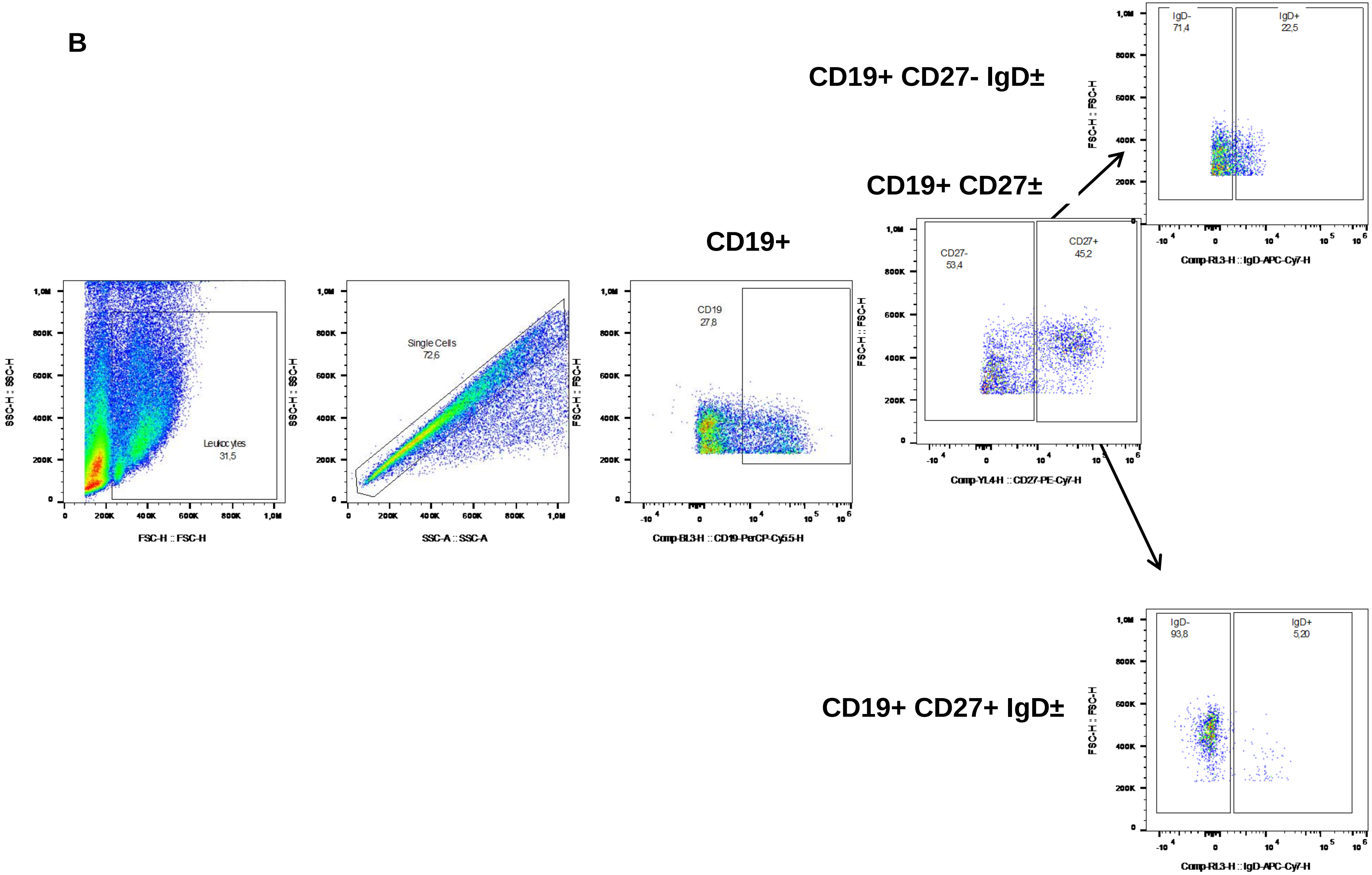

Disease Models & Mechanisms • Supplementary information

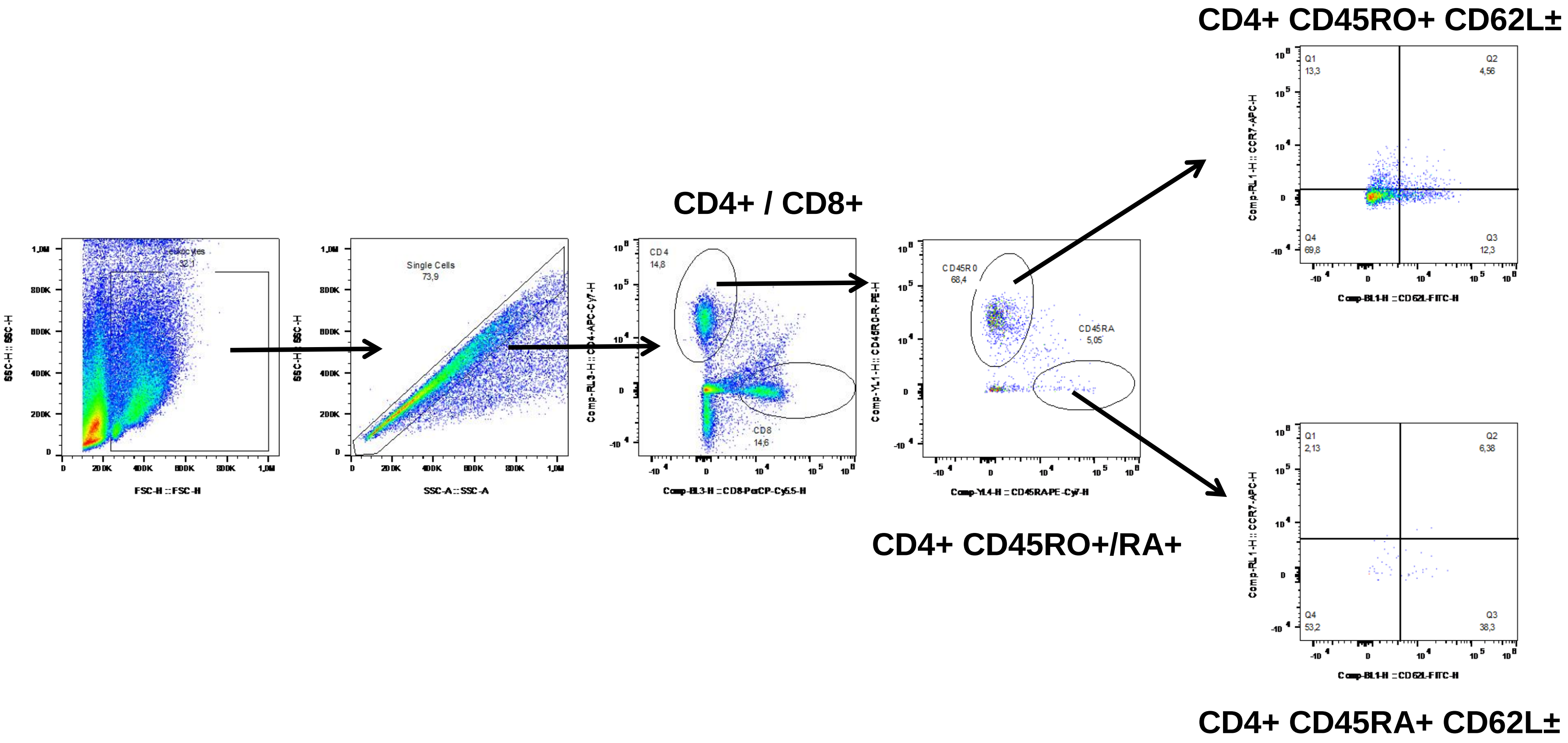

Disease Models & Mechanisms • Supplementary information

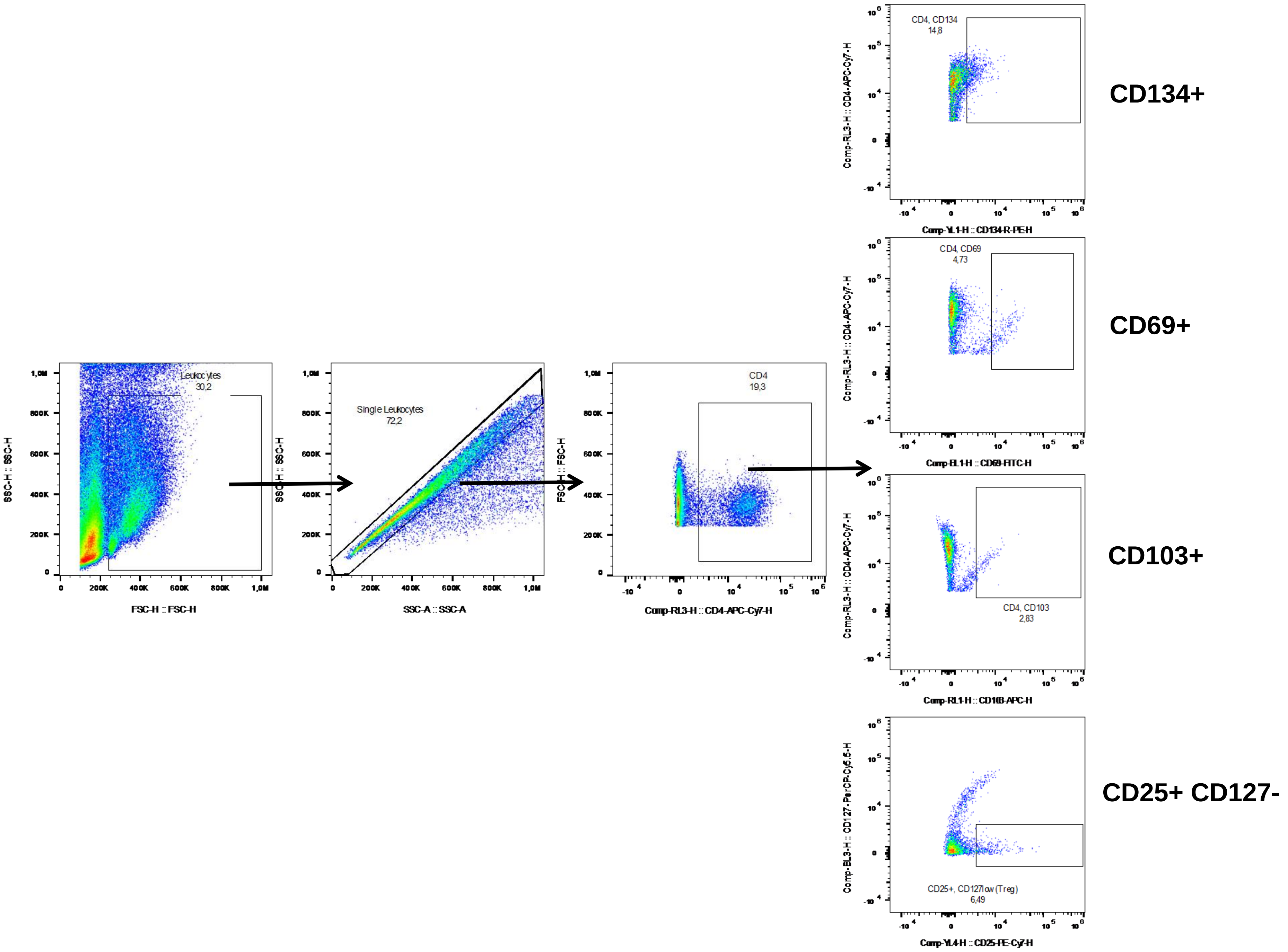
Leukocytes
30,2
SSC-H :: SSC-H
FSC-H :: FSC-H
Single Leukocytes
72,2
SSC-A :: SSC-A
CD4
19,3
Comp-RL3-H :: CD4-APC-Cy7-H
CD4, CD134
14,8
Comp-YL1-H :: CD134-R-PE-H
CD4, CD69
4,73
Comp-BL1-H :: CD69-FITC-H
CD4, CD103
2,83
Comp-RL1-H :: CD103-APC-H
CD25+, CD127low (Treg)
6,49
Comp-BL3-H :: CD127-PerCP-Cy5.5-H
Comp-YL4-H :: CD25-PE-Cy7-H
CD134+
CD69+
CD103+
CD25+ CD127-

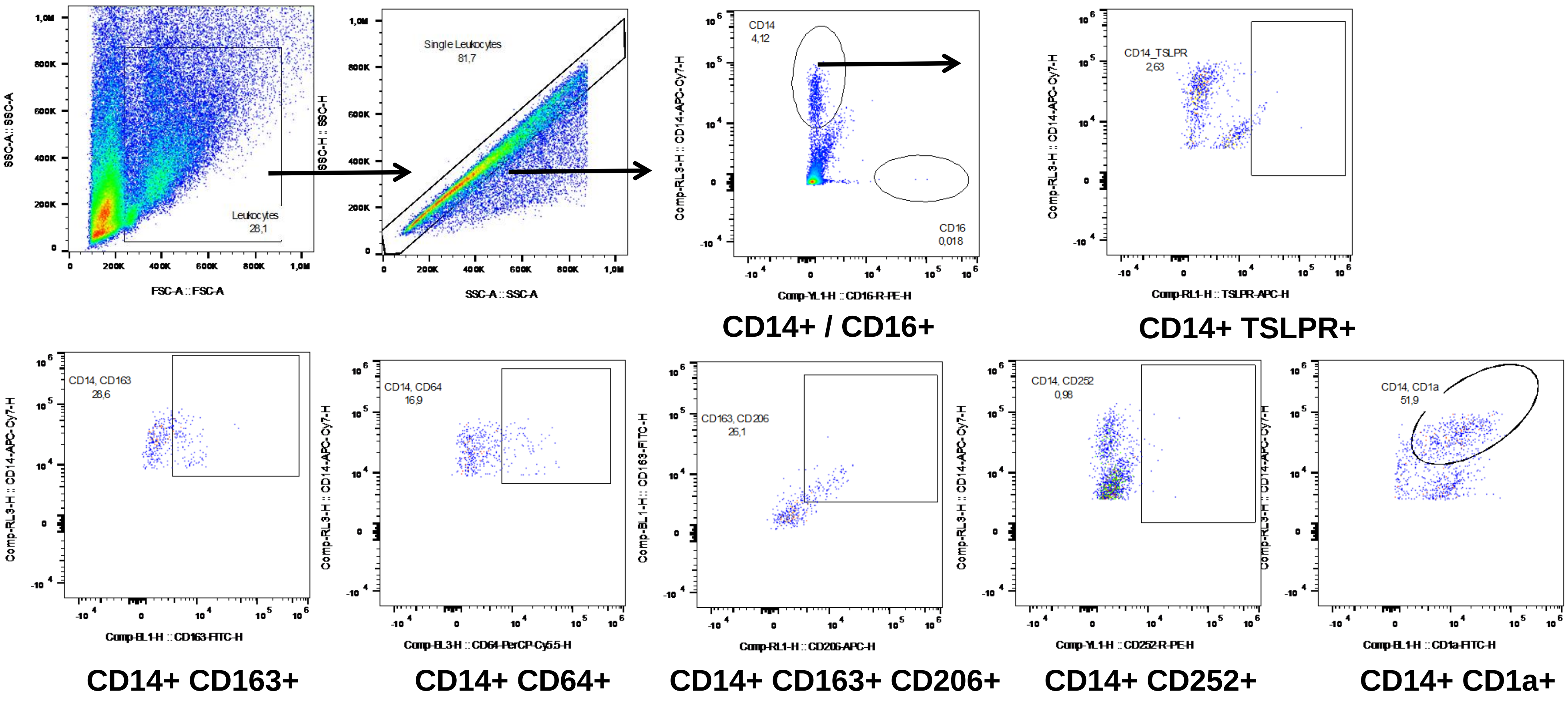
Leukocytes
28,1
FSC-A :: FSC-A
SSC-A :: SSC-A
Single Leukocytes
81,7
SSC-H :: SSC-H
CD14
4,12
CD16
0,018
Comp-YL1-H :: CD16-R-PE-H
Comp-RL3-H :: CD14-APC-Cy7-H
CD14+ / CD16+
CD14_TSLPR
2,63
Comp-RL1-H :: TSLPR-APC-H
CD14+ TSLPR+
CD14, CD163
28,6
Comp-BL1-H :: CD163-FITC-H
CD14+ CD163+
CD14, CD64
16,9
Comp-BL3-H :: CD64-PerCP-Cy5.5-H
CD14+ CD64+
CD163, CD206
26,1
Comp-BL1-H :: CD163-FITC-H
Comp-RL1-H :: CD206-APC-H
CD14+ CD163+ CD206+
CD14, CD252
0,98
Comp-YL1-H :: CD252-R-PE-H
CD14+ CD252+
CD14, CD1a
51,9
Comp-BL1-H :: CD1a-FITC-H
CD14+ CD1a+

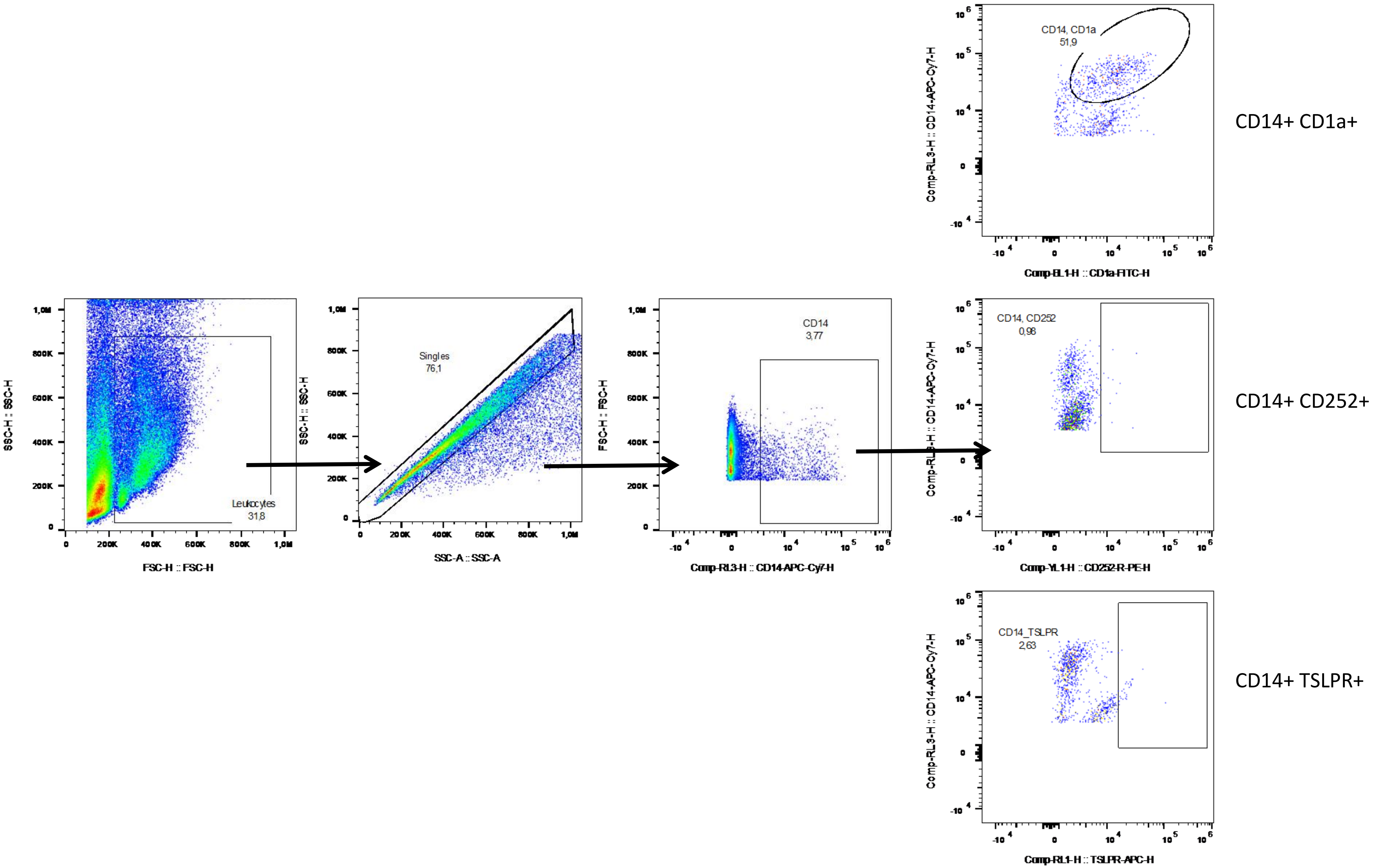

Disease Models & Mechanisms • Supplementary information

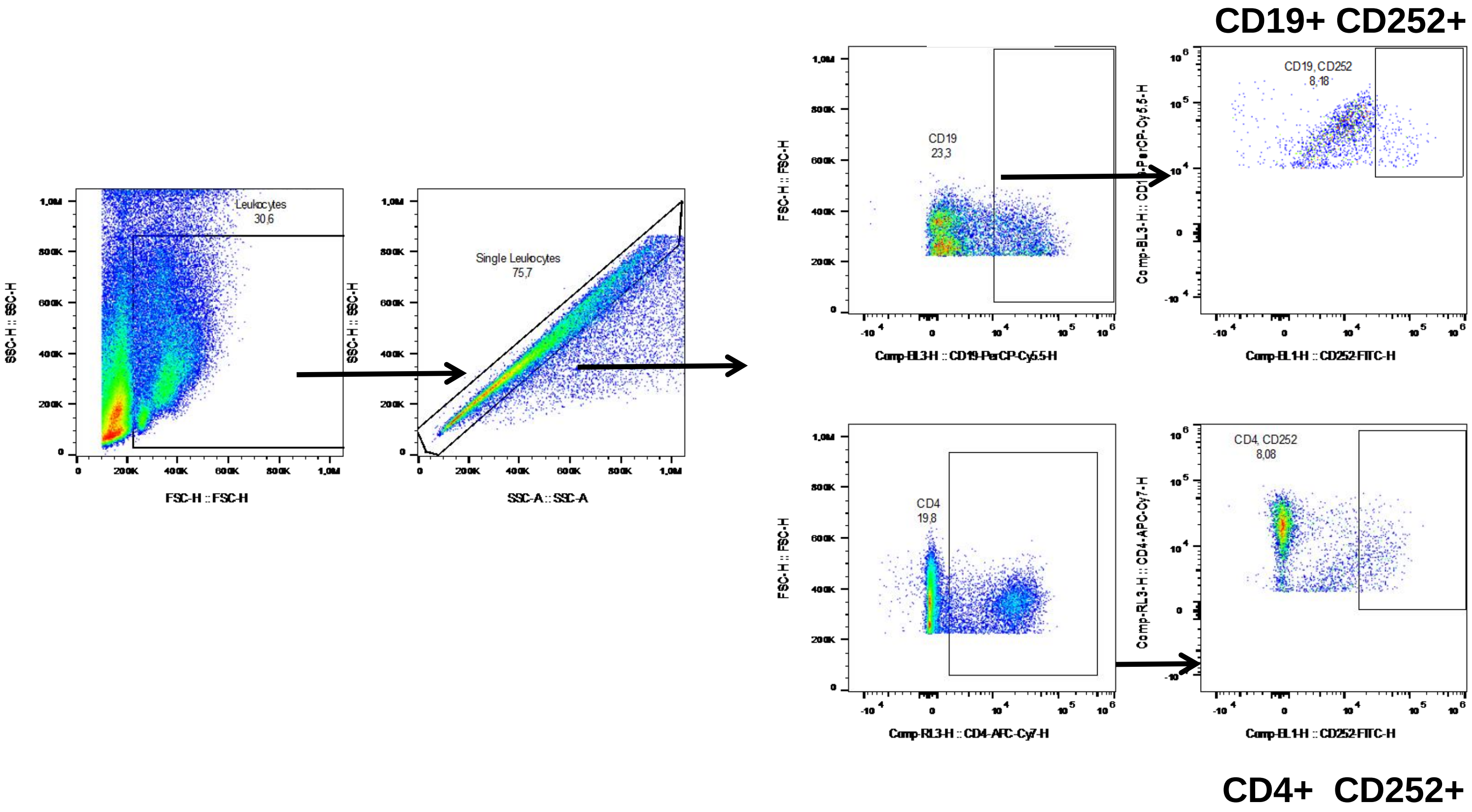

Figure S1 Gating Strategy. (A) human PBMC; (B) human leukocytes isolated from mouse spleen.

Disease Models & Mechanisms • Supplementary information

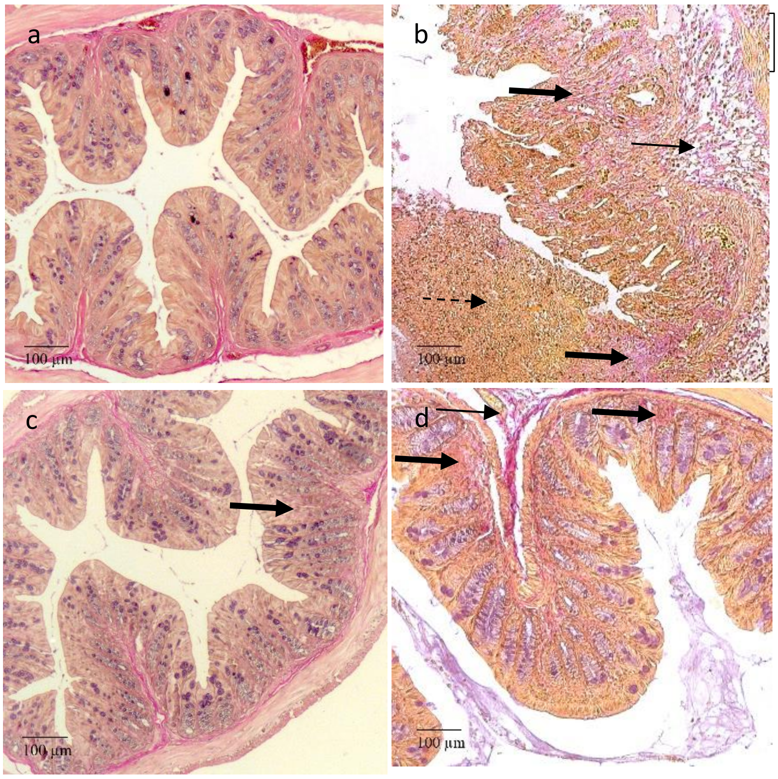

Figure S2. Comparison of the efficacy of oxelumab and adalimumab *in vivo*. Mice were treated as described in Figure 2. (A) Representative photomicrographs of Elastica-van-Gieson stained sections of distal parts of the colon from mice. (a) Unchallenged control, (b) ethanol challenged control treated with isotype, (c) ethanol challenged group treated with oxelumab, (d) ethanol challenged group treated with adalimumab. Arrows indicate edema and influx of inflammatory cells, dashed arrows indicate destruction of crypts and bold arrows fibrosis. (B) Histological scores depicted as boxplots. Boxes represent upper and lower quartiles, whiskers represent variability and outliers are plotted as individual points. For comparison of groups ANOVA followed by TukeyHSD was conducted. (0 '***' 0.001 '**' 0.01 '*' 0.05).

Disease Models & Mechanisms • Supplementary information

Table S1 Antibodies used to label human leukocytes

Surfacemarker	Colour	Cat # RRID #
CD19	Peridine-chlorophyll-protein complex cyanine dye (PerCP-Cy™ 5.5)	**BioLegend Cat# 302230, RRID:AB_2073119**
CD38	Phycoerythrin (PE)	**BioLegend Cat# 356603, RRID:AB_2561899**
CD27	Pe-Cy7	**BioLegend Cat# 356411, RRID:AB_2562257**
IgD	APC/Cy7	**BioLegend Cat# 348218, RRID:AB_11203722**
CD4	Allophycocyanin (APC)-Cy7	**BioLegend Cat# 317417, RRID:AB_571946**
CD45RA	PE/Cy7	**BioLegend Cat# 304126, RRID:AB_10708879**
CD45RO	PE	**BioLegend Cat# 304206, RRID:AB_314422**
CD62L	FITC	**BioLegend Cat# 304838, RRID:AB_2564162**
CCR7	APC	**BioLegend Cat# 353214, RRID:AB_10917387**
CXCR3	FITC	**BioLegend Cat# 353704, RRID:AB_10983066**
CCR6	PE/Cy7	**BioLegend Cat# 353405, RRID:AB_10918985**
CCR10	APC	**BioLegend Cat# 341505, RRID:AB_2291025**
CD8	PerCP-Cy™ 5.5	**BioLegend Cat# 344750, RRID:AB_2687201**
CD103	APC	**BioLegend Cat# 350215, RRID:AB_2563906**
CD14	APC-Cy7	**BioLegend Cat# 325619, RRID:AB_830692**
CD16	PE	**BioLegend Cat# 360703, RRID:AB_2562748**
TSLPR	APC	**BioLegend Cat# 322807, RRID:AB_2085327**
CD1a (biotin)/secondary Ab streptavidin	FITC	**BioLegend Cat# 300112, RRID:AB_389344**
CD64	PerCP-Cy™ 5.5	**BioLegend Cat# 305023, RRID:AB_2561585**
CD163	FITC	**BioLegend Cat# 333617, RRID:AB_2563093**
CD206	APC	**BioLegend Cat# 321109, RRID:AB_571884**
CD16	PE	**BioLegend Cat# 302007, RRID:AB_314207**
CD11b	APC-Cy7	**BioLegend Cat# 301341, RRID:AB_2563371**

Disease Models & Mechanisms • Supplementary information

CD11c	PE-Cy7	**BioLegend Cat# 301607, RRID:AB_389350**
CD69	FITC	**BioLegend Cat# 310903, RRID:AB_314838**
CD25	PE/Cy7	**BioLegend Cat# 302611, RRID:AB_314281**
CD134 (Ox40)	PE	**BioLegend Cat# 350003, RRID:AB_10641708**
CD127	Per CP Cy5	**BioLegend Cat# 351321, RRID:AB_10900253**
CD252	PE	**BioLegend Cat# 108805, RRID:AB_313404)**

Table S2 Cellular markers used to define immune cells

Marker	Definition
CD19+ CD27+ IgD+	Unswitched memory B-cell
CD19+ CD27+ IgD-	Switched memory B-cell
CD19+ CD38+	Plasma cell
CD4+ CD45RO+ CD62L- CCR7-	Effector memory CD4+ T- cell
CD4+ CD45RO+ CD62L+, CCR7+	Central memory
CD4+ CD103+	Mucosal regulatory CD4+ T cell
CD4+ CCR4+	Th2, effector regulatory T- cell
CD4+ CD25+ CD127-	Regulatory T-cell
Th1	CD4+ CXCR3+
Th2	CD4+ CCR4+ CCR6-
Th17	CD4+ CCR4+ CCR6+
Th22	CD4+ CCR4+ CCR6+ CCR10+
CD4+ CD134+	Activated CD4+ T cell
CD4+ CD69+	Activated CD4+ T cell
CD4+ CD25+	Activated CD4+ T cell
CD14+	MC*
CD14+ TSLPR+	MC, expressing TSLPR
CD14+ CD64+	M1MC, FcγR1 expressing
CD14+ CD163+ CD206+	M2 MC, scavenging cells
CD14+ CD1a	MC CD1a expressing
CD11b+	cDC1**
CD11b+ CD80/86+	cDC1, mature
CD11b+ TSLPR+	cDC1 TSLPR expressing
CD11b+ CD1a+	cDC1 CD1a expressing

*Monocyte

**Conventional dendritic cell

TSLPR: Thymic stromal lymphopoietin receptor

Disease Models & Mechanisms • Supplementary information

Table S3 Complete data set animal study

	Mean				SD				n			
**Leukocytes from spleen [% FoP]**	**Control**	**Isotype**	**Oxelumab**	**Adalimumab**	**Control**	**Challenge**	**Oxelumab**	**Adalimumab**	**Control**	**Challenge**	**Oxelumab**	**Adalimumab**
CD4+	9,33	19	22,99	17,63	4,65	14,4	15,54	13,96	6	24	18	21
CD4+ CD69+	17,4	28,72	27,68	25,91	5,74	12,68	11,89	11,54	5	24	18	21
CD4+ CD134+	47,22	39,29	31	33,58	30,31	27,1	23,67	23,41	6	24	18	21
CD4+ CD103+	16,51	3,85	6,84	4,63	39,72	2,77	4,93	6,53	6	24	18	21
CD4+ central memory	3,34	13,75	9,21	13,59	2,28	11,66	10,39	17,11	6	24	18	22
CD4+ effector memory	73,55	52,99	57,92	60,1	4,98	30,22	25,12	25,58	6	24	18	22
CD4+ effector	60,85	24,16	27,52	30,82	7,93	30,25	41,39	35,38	6	24	18	23
CD4+ naive	2,24	30,09	32,63	34,24	1,11	32,01	33,07	35,79	6	24	18	23
CD8+ effector memory	63,31	29,32	8,47	28,5	9,17	22,26	2,31	20,67	6	13	6	11
CD8+ central memory	0,41	3,62	12,18	9,86	0,34	7,66	17,09	18,13	6	19	12	17
CD14+	4,68	16,72	52,6	28,07	0,69	19,7	34,41	30,62	6	23	18	21
CD14+ CD64+	6,98	13,65	20,96	14,56	3,01	11,03	15,61	14,04	6	23	18	21
CD14+ CD163+	2,93	16,38	22,65	14,83	1,83	20,65	18,25	16,23	6	23	18	21
CD14+ CD163+ CD206+	1,91	9,68	12,93	7,53	2,52	12,25	12,97	9,06	6	18	12	15
CD14+ TSLPR+*	3,55	4,52	4,46	6,84	4,45	3,65	3,19	9,4	6	17	12	15
CD19+	11,66	11,73	12,7	14,26	3,6	3,24	2,96	5,38	6	24	18	22
CD19+ CD27+ IgD+	7,85	37,12	61,02	43,78	3,41	36,8	34,46	38,68	6	24	18	22
CD19+ CD27+ IgD-	90,35	54,78	33,37	49,98	3,75	38,89	33,48	40,27	6	24	18	22
**Scores**												
Clinical Score	2,33	3,11	0,94	1,45	0,51	3,25	0,72	1,14	6	26	18	24
Histological Score	0,16	4,08	1,58	2,08	0,4	2,3	2,23	1,69	6	24	17	24
Colon Score	0,33	2,75	0,55	0,87	0,51	1,7	1,42	1,32	6	24	18	24
**Amino Acids [µmol / l]**												
Glutamic Acid	60,25	100,79	117,96	91,61	19,46	21,13	19,65	24,36	6	21	17	19
Aspartic Acid	20,61	67,53	77,34	68,34	4,41	69,64	71,31	69,98	6	21	17	19
**Cytokines [pg/ml]**												
ms IL-1ß	4,23	12,53	14,3	15,12	2,41	12,2	10,6	19,64	6	19	11	17
ms IL-6	26,29	56,51	12,49	19,11	32,32	99,83	8,89	13,38	6	24	16	20
hu IFNγ		2,28	2,29	3,12		2,49	2,37	3,1		12	11	10
Hu IL-4	1,76	15,85	22,71	24,66	1,11	23,92	21,38	37,21	6	19	11	17

* Thymic stromal lymphopoietin receptor

	Δ		95% CI		Δ		95% CI		Δ		95% CI	
**Leukocytes from spleen [% FoP]**	**Isotype / Control**	**p-value**	**lower**	**upper**	**Oxelumab / Isotype**	**p-value**	**lower**	**upper**	**Oxelumab / Control**	**p-value**	**lower**	**upper**
CD4+	9,67				3,99				13,66			
CD4+ CD69+	11,32				-1,04				10,28			
CD4+ CD134+	-7,93				-8,29				-16,22			
CD4+ CD103+	-12,66				2,99				-9,67			
CD4+ central memory	10,41				-4,54				5,87			
CD4+ effector memory	-20,56				4,93				-15,63			
CD4+ effector	-36,69				3,36				-33,33			
CD4+ naive	27,85				2,54				30,39			
CD8+ effector memory	-33,99	0,003	-58,41	-9,57	-20,85				-54,84	6E-05	-83,4	-26,27
CD8+ central memory	3,21				8,56				11,77			
CD14+	12,04				35,88				47,92			
CD14+ CD64+	6,67				7,31				13,98			
CD14+ CD163+	13,45				6,27				19,72	0,09	-2,49	41,93
CD14+ CD163+ CD206+	7,77				3,25				11,02			
CD14+ TSLPR+*	0,97				-0,06				0,91			
CD19+	0,07				0,97				1,04			
CD19+ CD27+ IgD+	29,27				23,9				53,17	0,01	9,14	97,19
CD19+ CD27+ IgD-	-35,57				-21,41				-56,98	0,008	-102,33	-11,61
**Scores**												
Clinical Score	0,78				-2,17	0,0006	-3,21	-0,1	-1,39			
Histological Score	3,92	3E-04	1,5	6,32	-2,5	0,001	-4,16	-0,82	1,42			
Colon Score	2,42	2E-03			-2,2	4E-05	-3,38	-1	0,22			
**Amino Acids [µmol / l]**												
Glutamic Acid	40,54	8E-04	14,02	67,06	17,17				57,71	3,00E-06	30,51	84,91
Aspartic Acid	46,92				9,81				56,73			
**Cytokines [pg/ml]**												
ms IL-1ß	8,3				1,77				10,07			
ms IL-6	30,22				-44,02				-13,8			
hu IFNγ					0,01				2,29			
Hu IL-4	14,09				6,86				20,95			

* Thymic stromal lymphopoietin receptor

	Δ		95% CI		Δ		95% CI	
**Leukocytes from spleen [% FoP]**	**Adalimumab / Isotype**	**p-value**	**lower**	**upper**	**Oxelumab / Adalimumab**	**p-value**	**lower**	**upper**
CD4+	-1,37				-5,36			
CD4+ CD69+	-2,81				-1,77			
CD4+ CD134+	-5,71				2,58			
CD4+ CD103+	0,78				-2,21			
CD4+ central memory	-0,16				4,38			
CD4+ effector memory	7,11				2,18			
CD4+ effector	6,66				3,3			
CD4+ naive	4,15				1,61			
CD8+ effector memory	-0,82				20,03			
CD8+ central memory	6,24				-2,32			
CD14+	11,35				-24,53			
CD14+ CD64+	0,91				-6,4			
CD14+ CD163+	-1,55				-7,82			
CD14+ CD163+ CD206+	-2,15				-5,4			
CD14+ TSLPR+*	2,32				2,38			
CD19+	2,53				1,56			
CD19+ CD27+ IgD+	6,66				-17,24			
CD19+ CD27+ IgD-	-4,8				16,61			
**Scores**								
Clinical Score	-1,66	0,03	-3,21	-0,1	0,51			
Histological Score	-2	5E-03	-3,52	-0,47	0,5			
Colon Score	-1,88	1E-04			0,32			
**Amino Acids [µmol / l]**								
Glutamic Acid	-9,18				-26,35			
Aspartic Acid	0,81				-9			
**Cytokines [pg/ml]**								
ms IL-1ß	2,59				0,82			
ms IL-6	-37,4				6,62			
hu IFNγ	0,84				0,83			
Hu IL-4	8,81				1,95			

Disease Models & Mechanisms • Supplementary information

Table S4 Scores of individual mice

Maus	Donor	Treatment	Clinical Score	Colon Score	Histological Score
1	A	Isotype	1	2	3
2	A	Isotype	1	2	4
3	A	Isotype	3	3	3
4	A	Isotype	1	4	5
5	A	Isotype	1	1	5
6	A	Isotype	2	5	3
7	A	Adalimumab	0	1	2
8	A	Adalimumab	3	3	8
9	A	Adalimumab	1	0	4
10	A	Adalimumab	2	0	2
11	A	Adalimumab	2	1	2
12	A	Adalimumab	1	1	0
13	A	Oxelumab	2	0	0
14	A	Oxelumab	2	0	2
15	A	Oxelumab	1	0	0
16	A	Oxelumab	0	1	1
17	A	Oxelumab	2	6	7
18	A	Oxelumab	1	1	0
1	B	Isotype	0	1	5
2	B	Isotype	1	1	2
3	B	Isotype	1	1	3
4	B	Isotype	1	6	5
5	B	Isotype	8	6	11
6	B	Isotype	4	3	9
7	B	Adalimumab	0	0	0
8	B	Adalimumab	1	0	2
9	B	Adalimumab	1	0	2
10	B	Adalimumab	2	1	1
11	B	Adalimumab	2	2	1
12	B	Adalimumab	0	1	3
13	B	Oxelumab	0	0	0
14	B	Oxelumab	0	0	3
15	B	Oxelumab	1	1	0
16	B	Oxelumab	1	0	3
17	B	Oxelumab	1	0	0
18	B	Oxelumab	0	0	1
25	B	Isotype	13		
1	C	Isotype	0	2	5
2	C	Isotype	2	2	5
3	C	Isotype	5	2	3
4	C	Isotype	0	2	3
5	C	Isotype	2	2	2
12	C	Oxelumab	1	0	

13	C	Oxelumab	1	0	0
14	C	Oxelumab	1	0	0
15	C	Oxelumab	0	0	0
16	C	Oxelumab	2	1	5
17	C	Oxelumab	1	0	5
18	C	Adalimumab	1	0	1
19	C	Adalimumab	1	0	2
20	C	Adalimumab	1	0	2
21	C	Adalimumab	3	0	2
22	C	Adalimumab	1	0	2
23	C	Adalimumab	2	0	2
24	C	Isotype	8		
1	D	Isotype	4	3	5
2	D	Isotype	10	5	6
3	D	Isotype	3	6	3
4	D	Isotype	3	1	0
5	D	Isotype	2	3	3
6	D	Isotype	2	1	3
7	D	Isotype	3	2	2
8	D	Adalimumab	2	1	1
9	D	Adalimumab	1	1	1
10	D	Adalimumab	2	1	1
11	D	Adalimumab	0	1	1
12	D	Adalimumab	1	1	3
13	D	Adalimumab	5	6	5
14	D	Control	2	0	0
15	D	Control	3	0	0
16	D	Control	2	0	0
17	D	Control	3	0	1
18	D	Control	2	1	0
19	D	Control	2	1	0
